# Metabolic regulation is sufficient for global and robust coordination of glucose uptake, catabolism, energy production and growth in *Escherichia coli*

**DOI:** 10.1371/journal.pcbi.1005396

**Published:** 2017-02-10

**Authors:** Pierre Millard, Kieran Smallbone, Pedro Mendes

**Affiliations:** 1 MCISB, Manchester Institute of Biotechnology, University of Manchester, Manchester, United Kingdom; 2 School of Computer Science, University of Manchester, Manchester, United Kingdom; 3 LISBP, Université de Toulouse, CNRS, INRA, INSA, Toulouse, France; 4 Center for Quantitative Medicine and Dept. Cell Biology, UConn Health, Farmington, Connecticut, United States of America; The Pennsylvania State University, UNITED STATES

## Abstract

The metabolism of microorganisms is regulated through two main mechanisms: changes of enzyme capacities as a consequence of gene expression modulation (“hierarchical control”) and changes of enzyme activities through metabolite-enzyme interactions. An increasing body of evidence indicates that hierarchical control is insufficient to explain metabolic behaviors, but the system-wide impact of metabolic regulation remains largely uncharacterized. To clarify its role, we developed and validated a detailed kinetic model of *Escherichia coli* central metabolism that links growth to environment. Metabolic control analyses confirm that the control is widely distributed across the network and highlight strong interconnections between all the pathways. Exploration of the model solution space reveals that several robust properties emerge from metabolic regulation, from the molecular level (*e*.*g*. homeostasis of total metabolite pool) to the overall cellular physiology (*e*.*g*. coordination of carbon uptake, catabolism, energy and redox production, and growth), while allowing a large degree of flexibility at most individual metabolic steps. These properties have important physiological implications for *E*. *coli* and significantly expand the self-regulating capacities of its metabolism.

## Author summary

Metabolism is a fundamental biochemical process that enables cells to operate and grow by converting nutrients into ‘building blocks’ and energy. Metabolism happens through the work of enzymes, which are encoded by genes. Thus, genes and their regulation are often thought of controlling metabolism, somewhat at the top of a hierarchical control system. However, an increasing body of evidence indicates that metabolism plays an active role in the control of its own operation via a dense network of metabolite-enzyme interactions. The system-wide role of metabolic regulation is hard to dissect and so far remains largely uncharacterized. To better understand its role, we constructed a detailed kinetic model of the carbon and energy metabolism of the bacterium *Escherichia coli*, a model organism in Systems and Synthetic biology. Model simulations indicate that kinetic considerations of metabolism alone can explain data from hundreds of experiments, without needing to invoke regulation of gene expression. In particular, metabolic regulation is sufficient to coordinate carbon utilization, redox and energy production, and growth, while maintaining local flexibility at individual metabolic steps. These findings indicate that the self-regulating capacities of *E*. *coli* metabolism are far more significant than previously expected, and improve our understanding on how cells work.

## Introduction

Metabolism is a fundamental biochemical process that converts nutrients into energy and biomass precursors, thus enabling cells to maintain their structures, grow, and respond to their environment. While the topology of metabolic networks is fairly well known, understanding how metabolic behaviours emerge from the dynamic interactions of their molecular components remains one of the main challenges faced by systems biology and is crucial for the development of synthetic biology [1].

The operation of metabolic networks, *i*.*e*. the metabolic fluxes, represents the ultimate output of several regulatory mechanisms. Metabolic fluxes are functions of enzyme activities and of the concentrations of reactants, products, and other effectors. While the enzyme activities are the ultimate outcome of gene expression through the *hierarchy* of transcriptional, post-transcriptional, translational and post-translational regulatory mechanisms, the reactant and effector concentrations are directly regulated at the metabolic level by enzyme activities themselves. Hierarchical regulation, and in particular transcriptional regulation, has attracted much attention because of mature experimental methods, but also because of early examples of flux increase with enzyme induction [2, 3]. These studies suggested an intuitive picture where fluxes mainly depend on enzyme concentrations, themselves mainly dependent on the level of transcript—a view that puts genes and their regulation at the top of a hierarchy of control and that regards metabolism as mostly a consequence of gene expression. An increasing body of evidence, however, indicates that this view of a hierarchical (or “dictatorial”) regulation of metabolism by gene expression is too simplistic. Large-scale ^13^C-flux analyses revealed that flux distributions in *Saccharomyces cerevisiae* and *Escherichia coli* are incredibly robust to the deletion of global transcriptional regulators [4, 5]. Integration of transcript and enzyme abundances with fluxes measured under different environmental conditions indicated that hierarchical regulation is insufficient to explain most of the flux reorganizations [6–9]. Therefore metabolism can no longer be seen as a passive process primarily regulated at the hierarchical level, but rather that it plays an active role in the control of its own operation via a dense network of metabolite-enzyme interactions. However, because hundreds of these interactions simultaneously regulate fluxes, which in turn affect metabolite levels, the system-wide role of metabolic regulation is hard to dissect and so far remains largely uncharacterized.

Mathematical frameworks such as metabolic control analysis [10, 11] were developed to analyze such complexity and improve our understanding of the role of each interaction on the metabolic network. These frameworks are particularly useful when applied to (validated) kinetic models that quantitatively describe the mechanistic interactions between the molecular species and their dynamics. However, developing models that are truly representative of real cell metabolism requires large amounts of experimental data to establish complex rate laws and identify parameters for each interaction [12]. Most models have focused on single pathways or on small sub-systems [*e*.*g*. 13, 14–18]. Such models accurately predict the response of those pathways to perturbations and reveal insights on the role of particular regulatory interactions on the metabolic operation [19–21]. Great progress has recently been made to develop larger scale kinetic models using top-down approaches [22–29], hence paving the way towards comprehensive understanding of the role of metabolic regulation at the whole cell level. These large-scale kinetic models highlighted the system-wide impact of local properties on the functioning of metabolic networks, such as an improved metabolic flexibility caused by enzyme saturation [26]. However, these large scale models are typically constructed from whole-genome metabolic reconstructions using generic rate laws, and contain a low level of mechanistic details (in particular are mostly devoid of allosteric regulation). An alternative approach constructed a highly detailed model of an entire cell of *Mycoplasma genitalium* [30], but unfortunately while this model represents a considerably high level of mechanistic detail in many cellular processes, it entirely lacks metabolic regulation (as it uses dynamic flux balance analysis rather than a mechanistic kinetic model). Hence, many of the properties that emerge from metabolic regulation are not captured by current large-scale models.

In this study, we aim at investigating the role of metabolic regulation on the central metabolic network of *E*. *coli*, which constitutes the backbone of its metabolism by providing macromolecular precursors, reducing equivalents, and energy for growth and maintenance. While previous studies typically focused on the role of particular regulatory interactions, we attempt to determine whether more global and generic properties arise from the interplay of the many regulatory interactions that compose metabolic regulation. To accomplish this, a kinetic model of *E*. *coli* central carbon and energy metabolism was developed and validated against a large set of existing experimental data. This model includes more mechanistic details than previous ones, and the impact of metabolic regulation on this system was analyzed using local and global methods.

## Results

### Model development

**Fig 1:**
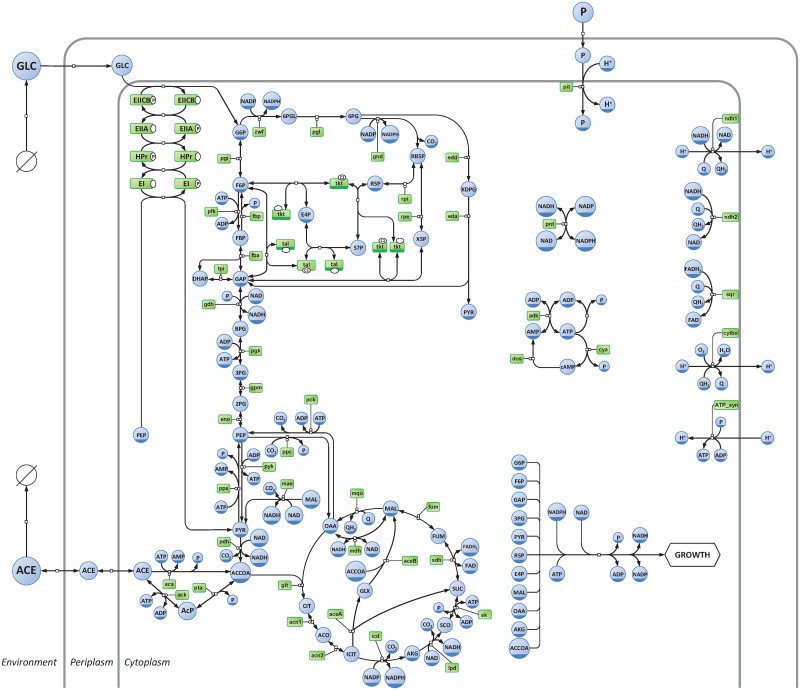
Representation of the central carbon and energy network of *Escherichia coli*. Metabolites and enzymes are shown in blue and green, respectively. The diagram adopts the conventions of the Systems Biology Graphical Notation process description [87].

The kinetic model developed in this study represents the central metabolism of *Escherichia coli* cultivated on glucose under aerobic conditions (Fig 1). This model contains 3 compartments (environment, periplasm and cytoplasm), 62 metabolites, and 68 reactions which represent the main central carbon and energy pathways of *E*. *coli*, namely: glucose phosphotransferase system (PTS), glycolysis and gluconeogenesis (EMP), pentose phosphate (PPP) and Entner-Doudoroff (EDP) pathways, anaplerotic reactions (AR), tricarboxylic acid cycle (TCA), glyoxylate shunt (GS), acetate metabolism (AC), nucleotide interconversion reactions (NC) and oxidative phosphorylation (OP). A reaction was also included to account for the consumption of metabolic precursors, reducing equivalents, and energy, and thus linking metabolism to cell proliferation. To account for metabolic regulation, a total of 255 metabolite-enzyme interactions (*i*.*e*. where metabolites modulate the reaction rates through thermodynamic or kinetic regulation, such as being substrates, products, allosteric modulators, or other type of inhibitors or activators) were included in the model, amongst which 34 are long-range regulatory interactions (*i*.*e*. where certain metabolites, which are not reactants, modulate the rates of these reactions).

Previously published kinetic models of *E*. *coli* metabolism were used as scaffolds to construct this model [18, 31, 32]. Both the number of pathways and the level of mechanistic detail were increased in the present model (S1 Table). For instance, this model now couples carbon metabolism with a detailed representation of oxidative phosphorylation, which makes it possible to balance the concentrations of cofactors (ATP/ADP/AMP, NAD(P)(H) and FAD(H_2_)) and simulate energy and redox metabolism. Previous models accounted for the consumption of metabolic precursors for growth in a decoupled way. This may be enough from the point of view of mass balance, but results in artifacts if used for an understanding of dynamics and regulation. In contrast the present model includes a single reaction to model growth, which ensures that the building blocks are consumed in stoichiometric proportions fixed by the cell composition, and not independently from each other. The rate of this reaction is a function of the intracellular concentrations of all the building blocks. This represents a significant improvement by satisfying the following growth rate properties: *i*) it monotonically increases with the availability of each building block, *ii*) it is asymptotically independent of each pool above a saturating concentration, and *iii*) it approaches zero if any pool approaches zero [21]. These properties were not reflected in the previous models [33].

The present model was calibrated to represent the metabolic state of *E*. *coli* cultivated under carbon limitation, a condition frequently experienced by this bacterium in laboratories, in industrial bioprocesses, and likely in its natural environment. To the extent possible, values of the biochemical parameters were taken from experimental determinations available in the literature. Parameters not available in the literature were estimated to reproduce steady-state and time-course experimental data obtained from a unique *E*. *coli* strain (the model strain K-12 MG1655) grown under a unique reference condition (M9 minimal medium with glucose as sole carbon source, dilution rate = 0.1 h^-1^, temperature = 37°C, pH = 7.0, pO_2_ > 20%) [13, 34–37]. This step is critical since both metabolite concentrations and fluxes depend on environmental conditions and differ between strains [38–40]. While results described below are largely in agreement with other experimental observations, *the model was not forced to reproduce them*, providing an important validation of the model. Detailed information on the construction and validation of the model is given in the Methods section and Supporting Information (S1 Text). The model is included in Supporting Information (S1 Model) formatted in SBML [41] and COPASI [42] formats, and is available from the BioModels database [43] with accession number MODEL1505110000.

### Metabolic regulation ensures robustness to fluctuations in enzyme levels and a high sensitivity to the environment

The control properties of *E*. *coli* central metabolism in the reference state (see above) were investigated under the metabolic control analysis framework [10, 11]. Flux ($C_{E}^{J}$) and concentration ($C_{E}^{M}$) control coefficients quantify the impact of a small change in the rate of each reaction (*e*.*g*. through change in the enzyme concentration *E*), on each flux (*J*) and each metabolite concentration (*M*). Since each metabolic step affects all fluxes and concentrations to some extent, we calculate a metric of its overall control on fluxes and concentrations as the L2 norm of all its flux- and concentration-control coefficients (see Methods), respectively.

**Fig 2:**
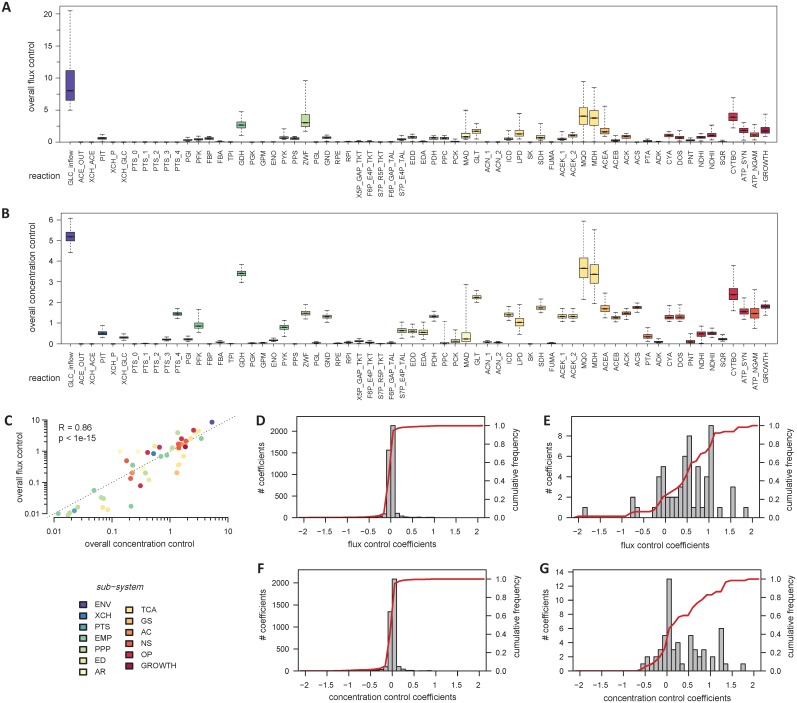
Control of *E*. *coli* metabolism. Overall control exerted by each reaction on fluxes (**A**) and metabolite concentrations (**B**). The overall flux and concentration control exerted by each reaction are correlated, as shown in panel **C**. Gray histograms show the distribution of flux control coefficients of enzymes (**D**) and environment (glucose supply reaction, **E**) on all the fluxes, and the distribution of concentration control coefficients of enzymes (**F**) and environment (**G**) on all the intracellular pools. Red lines (**D-G**) represent the cumulative frequency of each distribution.

The overall flux- and concentration-control by each step in the network is displayed in Fig 2. The main control point is the glucose inflow reaction with a control of 8.7 on fluxes and 5.3 on concentrations, using this summary metric. The system is therefore sensitive to its environment, as expected. Note that this is a *direct* sensitivity of metabolism to the environment, not through the (hierarchical) action of signal transduction and gene expression, which is not represented in this model; if it were its effect would thus be overlaid (likely with a delay) on the direct effect displayed in our model. Reactions that were identified by previous models as exerting a strong flux control under similar environmental conditions, such as the glucose phosphotransferase reactions or phosphofructokinase [13, 32, 44, 45], showed low control in our model (respectively 0.1 and 0.8). Rather, consistently with experimental evidence (see for example [46, 47–49]), the flux control was predicted to be shared between enzymes of all the pathways, amongst which cytochrome *bo* oxidase (reaction CYTBO, with 4.7 overall flux control), glucose-6-phosphate dehydrogenase (ZWF, 3.9), glyceraldehyde-3-phosphate dehydrogenase (GDH, 2.6), citrate synthase (GLT, 2.9) and the anabolic machinery (GROWTH, 1.4) are the ones with the largest share. A similar situation was observed for the control of concentrations, which is widely distributed across the network, and with the environment as the strongest control. In fact, a significant correlation (Pearson R = 0.86, P-value = 10^−15^) can be observed between the overall flux- and concentration-control exerted by each step (Fig 2C), indicating that, in general, enzymes which exert the strongest control on fluxes also exert the strongest control on concentration. A global sensitivity analysis [50] shows that these conclusions are robust with regard to parameter uncertainties (Fig 2A and 2B).

Further analysis confirmed the wide distribution of flux control across all the enzymes, with 97% of the individual control coefficients between -0.3 and 0.3 (Fig 2D). These observations, in agreement with the view that “rate-limitation” is distributed across the network and is variable [51], explain why fluxes are robust to moderate or even large changes of enzyme levels. The fluxes in this reference state are more sensitive to the environment, with 75% of the control coefficients exerted by the glucose supply reaction higher than 0.3 (Fig 2E). Similar conclusions were reached regarding the control of metabolite concentrations, which is distributed across the network (Fig 2B), with 97% of the control coefficients exerted by enzymes between -0.3 and 0.3 (Fig 2F) and 49% of the control coefficients exerted by the glucose supply reaction higher than 0.3 (Fig 2G).

### The distributed control results in strong interconnections between all the pathways

**Fig 3:**
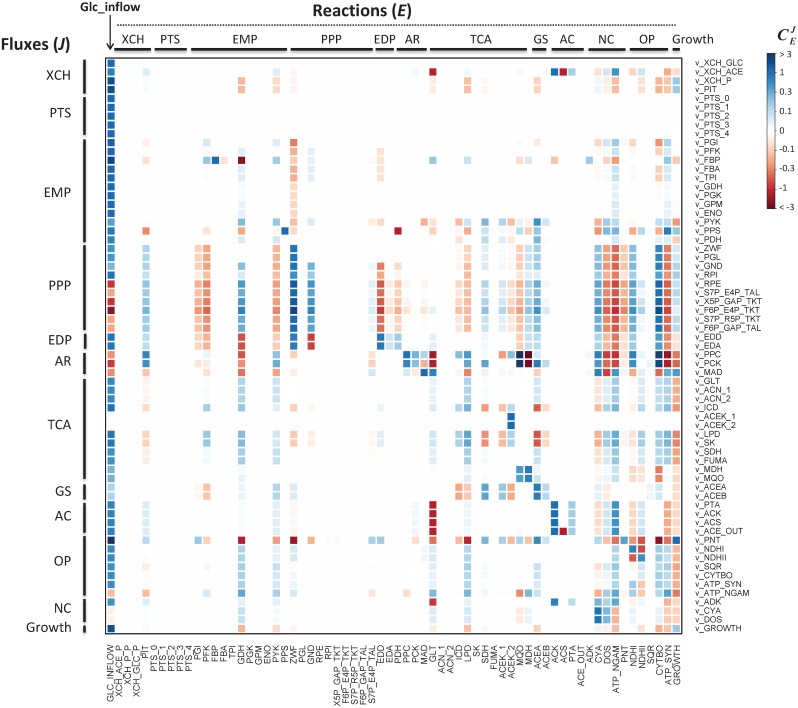
Heatmap of individual flux control coefficients. Columns represent the controlling reactions and rows represent the fluxes under control. Red and blue colors represent negative and positive values of flux control coefficients, respectively, and color intensity indicates strong (darker) to low (lighter) control.

Despite the low control exerted by enzymes over fluxes and concentrations at the network level, a detailed analysis of flux control coefficients reveals generic regulatory patterns between most of the pathways (Fig 3). A general observation is that the control of each pathway resides largely outside of itself.

For example, the control of the partition of carbon through competing pathways is shared between enzymes of each pathway. The glycolytic phosphofructokinase (PFK) exerts a (small) negative control on the PPP and ED fluxes ($C_{PFK}^{ZWF}=-0.15$) and a positive control on the glycolytic flux ($C_{PFK}^{PGI}=0.05$), while the glucose-6-phosphate dehydrogenase (ZWF) of the PPP and ED pathways exerts a strong positive control on its own flux ($C_{ZWF}^{ZWF}=0.75$) and a negative control on the glycolytic flux ($C_{ZWF}^{PFK}=-0.12$). Similar behavior is observed at the main metabolic branch nodes, *e*.*g*. between the TCA cycle and the glyoxylate shunt or between the pentose phosphate and Entner-Doudoroff pathways. It is important to note that the fraction of flux diverted to each branch does not depend only on the local enzyme kinetics, contrary to what is sometimes suggested [6], but on several enzymes of each of the competing pathways.

Several feedforward and feedback interactions are also observed between the pathways. For instance, the pyruvate kinase (PYK) controls fluxes through the TCA cycle ($C_{PYK}^{SDH}=0.07$), and is controlled by some TCA reactions ($C_{LPD}^{PYK}=0.06$,$\;C_{SDH}^{PYK}=0.19$). Similarly, the ATP demand (that can be represented by the ATP utilization for maintenance, ATP_NGAM) is activated by glycolysis ($C_{GDH}^{ATP\_NGAM}=0.26$) and exerts in turn a positive feedback control on this pathway ($C_{ATP\_NGAM}^{PGI}=0.12$) and a negative control on growth ($C_{ATP\_NGAM}^{GROWTH}=-0.09$), as observed *in vivo* [72]. Interestingly, biomass synthesis (GROWTH) is strongly controlled by the upstream glucose supply ($C_{GLC\_INFLOW}^{GROWTH}=1.5$), with all other control coefficients lower than 0.13. In turn, biomass synthesis exerts a small but global feedback control on most catabolic fluxes.

Those several, intertwined feedback and feedforward interactions stress the high degree of functional organization of the central carbon and energy metabolism. This may be an important feature to maintain the coordination between the different pathways at the cellular level: if the rate of a particular reaction—be it upstream or downstream—is affected by a perturbation, this information will be transmitted from this “sensor reaction” to the entire system, resulting in a global response. Note that this response is sensed in a very short time scale, rather than the slower response that happens after signal transduction and consequent changes in gene expression.

### Metabolic regulation couples growth to glucose uptake

To get a broader picture of the role of metabolic regulation on the coordination of *E*. *coli* metabolism, the solution space of this network was explored with and without considering metabolic regulation. Two versions of the model were used: the kinetic version which accounts for metabolic regulation, and a stoichiometric version of the same model which contains only stoichiometric constraints (and is thus similar to a flux balance analysis model). The solution space of each model was explored using a random sampling approach: 600,000 flux distributions were uniformly sampled from the solution space using the stoichiometric model, and steady-states were simulated for 600,000 sets of random enzyme levels using the kinetic model. For each set, enzyme levels (*i*.*e*. *Vmax*) were sampled from a log uniform distribution (between 0.1 and 10 times the enzyme levels of the initial model) to ensure each order of magnitude to be sampled in similar proportions. It is important to mention that cells do not express enzymes levels according to the distribution generated, therefore the distribution of the variables is not expected to provide any information on the probability for a cell to reach a specific state *in vivo* [50]. Rather, uniformity is used to clearly grasp the functional implications of applying metabolic regulation to the network.

**Fig 4:**
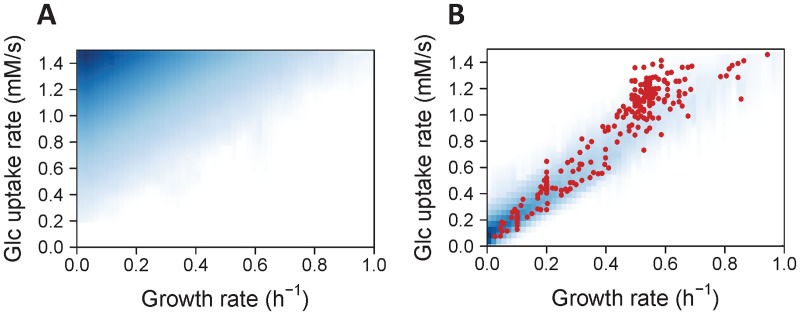
Exploration of the solution space of *E*. *coli* metabolism with and without metabolic regulation. Density of scatter plots between glucose uptake and growth rates sampled using the stoichiometric model (**A**, without metabolic regulation) or the kinetic model (**B**, with metabolic regulation). Shades of white to blue denote null to high frequency, respectively. Red dots are measurements obtained from 254 independent cultivations of wild-type and mutant *E*. *coli* strains on glucose, carried out under a wide range of cultivation conditions.

We first investigated the relationship between supply (glucose uptake) and demand (growth), which provides information on the allocation of resources by the metabolic network [52]. Direct sampling of the solution space (Fig 4A) revealed that most of the metabolic states are not efficient in term of resource allocation: most of them correspond to a high glucose uptake rate, but with a low growth rate, because this situation significantly increases the attainable intracellular flux states. Interestingly, the opposite picture is observed when metabolic regulation is applied on this network (Fig 4B): a smaller region of the solution space is reached, where the growth rate is now coupled to the glucose uptake rate.

To evaluate this prediction quantitatively, we gathered from the literature experimental data obtained from 254 growth experiments carried out under similar environmental conditions (glucose as sole carbon source in aerobic conditions) [5, 27, 34, 53–67] (S2 Dataset). These data were collected from “wild-type” and mutant strains obtained by deletion or overexpression of central metabolic enzyme genes or global regulators of gene expression, and cultivated under a wide range of experimental conditions. Therefore, these 254 data represent a very broad range of the metabolic states that can be expressed by *E*. *coli* growing on glucose. Importantly, these data were *not* used for parameter estimation, thereby they constitute an independent validation and provide a robust assessment of the predictive ability of the model. These experimental observations correspond to the region of the solution space less frequently sampled using the stoichiometric model, but they closely match the region sampled by the kinetic model (Fig 4B). This means that the observed physiology of *E*. *coli* is closer to the metabolic model that is regulated by metabolite-enzyme interactions (the kinetic model) than it is to a metabolic model that would be regulated by gene expression alone (the stoichiometric model). Hence, metabolic regulation alone, *without needing to invoke coordinated expression of genes*, seems to be sufficient to explain the emergence of a coupling between anabolic (growth) and catabolic (glucose uptake) fluxes, and thereby appears to be a major determinant of the overall cellular physiology by ensuring an efficient and robust allocation of nutrients towards growth.

### Metabolic regulation coordinates several processes at the cellular level while maintaining flexibility

**Fig 5:**
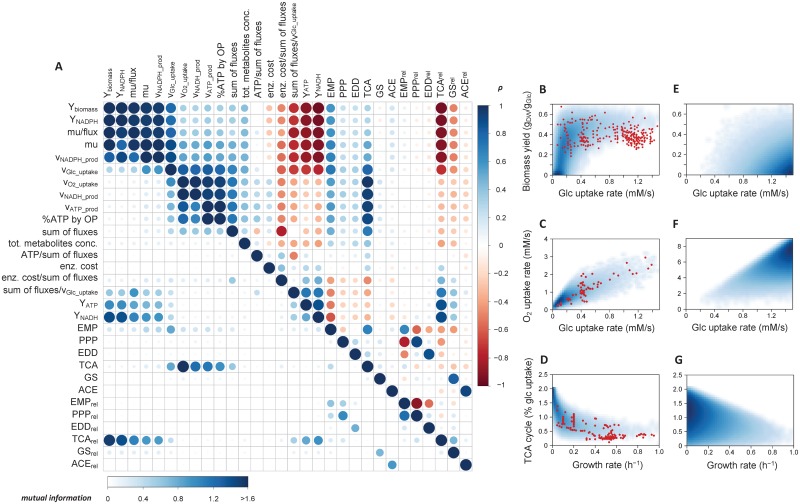
Identification of the functional couplings that are independent of enzyme levels. Steady-states were simulated for 600,000 sets of random enzyme levels, and the relationships between various systemic variables, absolute fluxes, and relative fluxes through the different pathways were identified using Spearman correlation test (above diagonal) and mutual information (below diagonal). For Spearman correlation test, red and blue colors represent negative and positive correlations, respectively, and color intensity and circle size indicates high (darker, larger) to low (lighter, smaller) correlation coefficient. For mutual information, color and circle size denote low (white, smaller) to high (blue, larger) mutual information, respectively. The density of scatter plots between particular steady-state variables (glucose and oxygen uptake, growth rate, biomass yield, and relative fluxes through the TCA cycle) sampled using the kinetic model (**B-D**, with metabolic regulation) or the stoichiometric model (**E-G**, without metabolic regulation). Shades of white to blue denote null to high frequency, respectively. Red dots are measurements obtained from a total of 266 independent cultivations (254, 65 and 192 in panels **B**, **C** and **D**, respectively) of wild-type and mutant *E*. *coli* strains on glucose, carried out under a wide range of cultivation conditions.

We extended the above analysis to determine whether additional couplings emerge from metabolic regulation. Several variables representative of the physiological state of *E*. *coli* were calculated for each steady-state reached by the kinetic model, namely: growth and glucose uptake rates, ATP, NADH and NADPH production rates, sum of all intracellular fluxes, sum of all intracellular metabolite concentrations and cost of enzymes (defined as the product of enzyme concentration and number of amino acids of the corresponding enzyme, summed over all reactions, as detailed in Methods). Additional variables derived thereof were also computed: biomass, ATP, NADH and NADPH yields, enzyme cost and ATP production rate per sum of fluxes, and sum of fluxes per glucose consumed. Pairwise relationships between systemic variables and (absolute and relative) fluxes through the main pathways were examined using Spearman correlation and mutual information. The outcome is a correlation matrix which maps the degree of functional coupling between all the variables (Fig 5A). The same patterns were highlighted by both methods, which indicate that these couplings are monotonic since mutual information, but not Spearman correlation, would identify non-monotonic relations.

A systematic positive correlation was predicted between some anabolic and catabolic fluxes and yields: glucose uptake rate, growth rate, NADPH production rate, biomass yield and NADPH yield (with ρ > 0.85), indicating a considerable degree of coordination in the metabolic operation. These variables are negatively correlated with the energetic (ATP and NADH) yields (ρ < -0.85), which is consistent with the fact that the single carbon source, glucose, is used by two competing metabolic processes: energy production and biomass synthesis, reflected in an increase in Y_ATP_ and increase in biomass yield, respectively. The oxygen uptake rate also correlated positively with ATP and NADH production (with ρ > 0.90), reflecting the important role of oxidative phosphorylation in energy production under aerobic conditions.

The low correlation coefficient between the sum of fluxes and the cost of enzymes (ρ = 0.11) indicates that the sum of fluxes cannot be considered as a proxy for enzyme investment *per se*. The outcome of predictive analyses based on this assumption (such as the minimization of the sum of fluxes in FBA according to the hypothesis that cells minimize their enzyme levels) should therefore be interpreted with caution.

In general, systemic variables correlated poorly with relative and absolute fluxes from most of the pathways. This is interesting as it shows that while there is coordination between several processes, there is nevertheless a significant degree of flexibility in the intracellular flux distribution. A notable exception was observed for the TCA cycle: its absolute flux is positively correlated with catabolic and energy fluxes (v_O2_uptake_, v_Glc_uptake_, v_NADH_production_, v_ATP_production_, with ρ > 0.84), and the relative contribution of this pathway negatively correlated with anabolic rates and yields (growth and NADPH production rates, ρ < -0.92) and positively correlated with energy yields (ρ > 0.90 for ATP and NADH yields). Thus, the partition of carbon between energy production (ATP and NADPH) and growth (via the synthesis of many anabolic precursors) is predicted to be realized primarily at the level of the TCA cycle and appears to be largely controlled at the metabolic level.

To evaluate these model predictions, additional experimental data on extracellular and intracellular fluxes (growth rate, glucose and oxygen uptake rates, and TCA cycle fluxes through the citrate synthase) were collected from the literature [5, 27, 34, 53, 54, 56, 58–60, 62–65, 67] (S2 Dataset). These data, which were not used to calibrate the model, covered the particular regions highlighted by the kinetic model (Fig 5B–5D). The excellent agreement between the spread of simulated and experimental data strongly supports the existence of the functional couplings predicted by the model. It is important to mention that these couplings are *not* caused by stoichiometric constraints since they are *not* observed when the solution space is uniformly sampled using the stoichiometric model (Fig 5E–5G). The results also show that the coordination of gene expression by hierarchical regulatory mechanisms is not an important factor in these couplings since they are still maintained when enzyme levels are changed randomly. In contrast, metabolic regulation brought about by metabolite-enzyme interactions is sufficient to explain their emergence; therefore they represent intrinsic properties of the central metabolism of *E*. *coli*.

**Fig 6:**
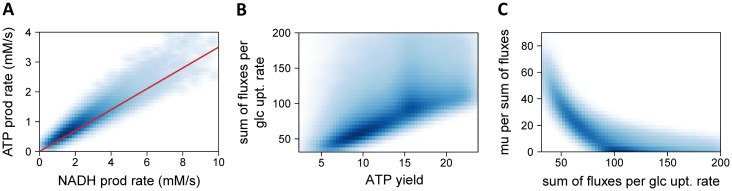
Some functional couplings observed in different microorganisms are predicted by the model. Predicted couplings between the ATP and NADH production rates (**A**), between the sum of fluxes per glucose uptake rate and the ATP yield (**B**), and between the growth rate per sum of fluxes and the sum of fluxes per glucose uptake rate (**C**). Shades of white to blue denote null to high frequency, respectively. The red line in panel A corresponds to the linear correlation proposed by [73] from energy production fluxes estimated using ^13^C-flux data.

Interestingly, additional couplings predicted by the model were recently observed *in vivo* in both prokaryotic (*E*. *coli*) and eukaryotic (*S*. *cerevisiae*) microorganisms [73]: between the ATP and NADH production rates (ρ = 0.93, Fig 6A), between the sum of fluxes per glucose uptake rate and the ATP yield (ρ = 0.71, Fig 6B), and between the growth rate per sum of fluxes and the sum of fluxes per glucose uptake rate (ρ = -0.85, Fig 6C). Since the central metabolic networks of *E*. *coli* and *S*. *cerevisiae* are highly conserved, the present results may explain why similar properties are observed in both microorganisms, though this hypothesis requires further investigation.

### Metabolic regulation does not significantly shrink the solution space defined by the stoichiometric constraints

**Fig 7:**
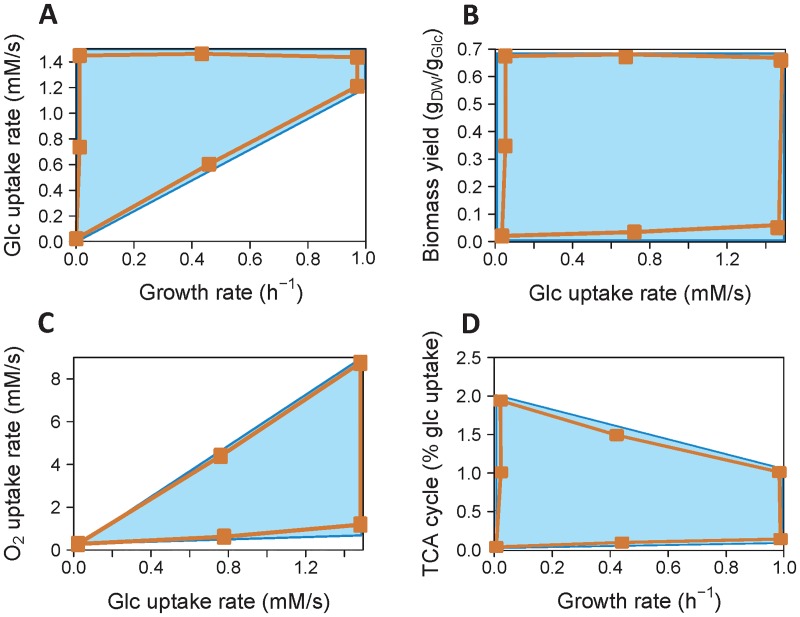
Boundaries of the solution space with and without metabolic regulation. The solution space defined only by stoichiometric constraints (blue area) was computed using the stoichiometric model. The solution space defined when metabolic regulation is taken into account (orange area) was estimated using the kinetic model, by optimizing enzyme levels with particular metabolic states (orange dots) as objective functions. The two solution spaces are similar, indicating that metabolic regulation does not significantly shrink the solution space, at least for the variables investigated here (**A**, glucose uptake rate vs. growth rate; **B**, biomass yield vs. glucose uptake rate; **C**, oxygen uptake rate vs. glucose uptake rate; **D**, TCA cycle flux—relative to glucose uptake—vs. growth rate).

The results presented above support the view that metabolic regulation reduces the solution space defined by the stoichiometric constraints, as previously suggested [68, 69]. However, the very low probability regions of the solution space might not be captured by random sampling approaches [50]. To test further if metabolic regulation actually shrinks the solution space of *E*. *coli* central metabolism, its boundaries were determined with and without considering metabolic regulation by using the kinetic and the stoichiometric models, respectively. Unexpectedly, the boundaries were similar for both models (Fig 7). This indicates that metabolic regulation does not shrink the solution space of the system—and thus does not restrict the metabolic capabilities of *E*. *coli*–, at least for the variables considered here. Thus we have to conclude that evolution must have “discovered” this region of parameter space which gives selective advantage to the organism.

### Metabolic regulation maintains global metabolite homeostasis

**Fig 8:**
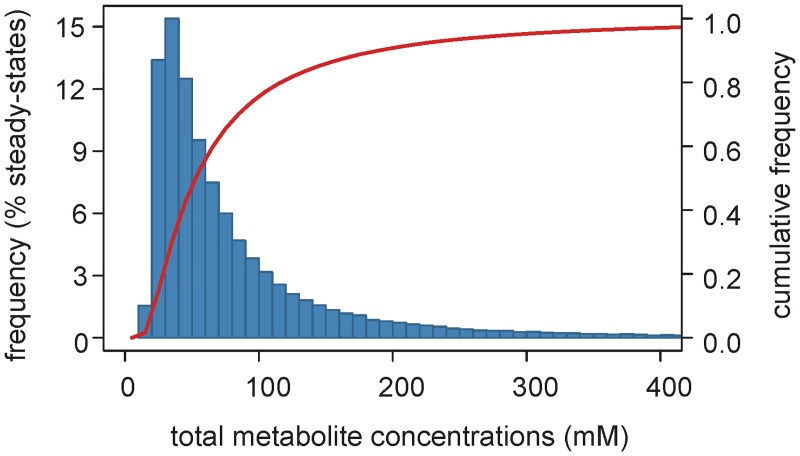
Metabolite homeostasis is largely driven my metabolic regulation. Distribution (blue bars) and cumulative frequency (red line) of the total concentrations of intracellular metabolites for steady-states simulated from 600,000 random enzyme levels.

It has been shown that metabolic regulation plays an important role in metabolite homeostasis, which prevents osmotic stress and disadvantageous spontaneous reactions by avoiding large changes in metabolite concentrations (for example see [20, 70]). Interestingly, we noticed that, for 86% of the steady-states reached by the kinetic model (when the enzyme levels were chosen at random), changes in total concentration of metabolites are lower than three-fold relative to the calibrated state (47 mM) (Fig 8), but the changes in fluxes are several orders of magnitude higher. This narrow range of predicted intracellular concentrations is physiologically relevant [63, 71]. Since no constraints on metabolite concentrations were included in the model, we conclude that metabolic regulation alone may explain global metabolite homeostasis, while still allowing significant changes in fluxes.

## Discussion

In this study, we investigated the contribution of metabolic regulation on the operation of the central metabolism of *E*. *coli*, which provides building blocks, cofactors, and energy for growth and maintenance. We developed, to our knowledge, the first detailed kinetic model of this system that links metabolism to environment and cell proliferation through intracellular metabolites levels. This model, validated by 778 independent flux data from some 266 experiments, allowed the identification of several properties which emerge from metabolic regulation and explain many experimental observations of *E*. *coli*’s physiology. The intrinsic, self-regulating capacities of *E*. *coli* central metabolism appear to be far more significant than previously expected. The results presented here imply that gene regulation is not required to explain these properties.

Metabolic control analysis showed that the flux and concentration control exerted by single enzymes is low and largely distributed across the network, confirming again the insights of Kacser and Burns [51]. This significantly contrasts with the outcome of previous kinetic models [13, 32, 44, 45], where a few enzymes were predicted to exert most of the flux control, but is in line with much experimental evidence [7, 21, 46, 47, 72]. Our results therefore support the view that the concept of “rate-limiting” steps does not apply to *E*. *coli* metabolism, and likely not to the metabolism of other organisms. Its persistence in the literature is a major handicap to understanding metabolism. In fact, the central metabolism is not even self-contained in terms of control due to a large portion of control being exerted by the environment, making *E*. *coli* responsive to environmental changes. One of the most striking examples of this phenomenon is manifested in growth controlling most fluxes but being controlled virtually by glucose availability alone. The low control exerted by single enzymes on the system makes the metabolic operation of *E*. *coli* robust to fluctuations of enzyme levels that may arise from noise in gene expression or other factors. Moreover, the majority of control resides not within but outside the controlled pathways. The dense, yet highly organized, interactions between pathways allow a rapid and coordinated response of the entire system to perturbations.

Exploration of the solution space indicated that metabolic regulation does not significantly restrict the metabolic capabilities of *E*. *coli*, as was previously believed [68, 69]. While the observed behavior of many different *E*. *coli* strains and mutants are confined to a small region of the solution space, this is not due to kinetic constraints as it is possible to simulate other behaviors simply by changing parameter values. This apparent paradox can be resolved, of course, if the action of natural selection had favored these behaviors.

The systematic mapping of the relationships between various systemic variables revealed that metabolic regulation is sufficient to explain the emergence of several functional couplings, which are independent from gene regulation (since they are conserved when enzyme levels are changed randomly by orders of magnitude) and cannot be explained by stoichiometric constraints. An important finding is that metabolic regulation alone may be responsible for the coordination of major catabolic, energetic and anabolic processes at the cellular level to optimize growth. Metabolic regulation thus appears to be sufficient to maintain multi-dimensional optimality of *E*. *coli* metabolism [65]. Despite this overall coordination, there is a large degree of flexibility at most individual metabolic steps. The role of metabolic regulation in maintaining global homeostasis of intracellular metabolite pools under a broad range of flux states was also verified by the present model. The modeling results were in excellent agreement with experimental data, even quantitatively. *E*. *coli* metabolism displays remarkably robust yet simple emergent properties, and these properties have major implications on its overall cellular physiology, *e*.*g*. by preventing unnecessary osmotic stress, maintaining the coordination between key processes, and optimizing the allocation of resources towards particular functions such as growth.

The self-regulating capabilities of *E*. *coli* central metabolism reflect the evolutionary selection that has been exerted on the ensemble of enzymes (in terms of kinetic and regulatory properties, but not necessarily of expression levels) to realize a network with these properties. Since central metabolism is essential in most organisms and is highly conserved across the three domains of life, it is tempting to speculate that metabolic regulation is responsible for the very similar operation principles observed in different organisms [73].

Of course we do not suggest that hierarchical regulation does not play an important role in the metabolic operation of *E*. *coli*, but it is in addition to the properties observed here, since these can operate without it. For instance, the robustness of the flux partition to the deletion of global transcriptional regulators was interpreted as a low control of this partition at the hierarchical level [5], and our results confirm that this robustness lies, to some extent, in metabolic regulation, given the low control exerted by enzymes. However, this conclusion is valid only for moderate changes of enzyme levels (with the notable exception of the flux through the TCA cycle), and other mechanisms (such as hierarchical regulation) are required to explain the robust flux partition. Expanding the kinetic model to incorporate regulation of gene expression will be needed ultimately to understand the interplay between these two regulatory levels [19, 74, 75, 88].

## Materials and methods

### Model construction

The kinetic model of the central carbon and energy metabolism of *Escherichia coli* K-12 MG1655 (Fig 1) was developed with the software COPASI (build 45) [42]. This model is briefly described in this section, and additional information can be found in Supporting Information (S1 Text). The model is available in SBML and COPASI formats in Supporting Information (S1 Model), as well as from the Biomodels database [43] with identifier MODEL1505110000.

#### Compartments

The model contains three compartments: the environment and the two cellular compartments: periplasm and cytoplasm. The periplasmic volume is set to be 20% of the total cell volume [76]. Since the calibrated model simulates the metabolic operation of *E*. *coli* cultivated under glucose-limited conditions (chemostat), two reactions were included to represent the continuous glucose inflow into the environment and acetate wash out (GLC_FEED and ACE_OUT, respectively).

#### Exchange reactions

Biological systems are open systems, three exchange reactions were therefore included to enable the transport of extracellular glucose (XCH_GLC), acetate (XCH_ACE) and phosphate (XCH_P) between the environment and the periplasm. Their rate was modelled as a saturable, porin-facilitated diffusion process through the outer membrane [77], using a reversible Michaelis-Menten kinetics.

#### Central carbon metabolism

Detailed description of the glucose phosphotransferase system was taken from [18]. Reactions of glycolysis, TCA cycle, glyoxylate shunt, and pentose phosphate and Entner-Doudoroff pathways were taken from [32]. Inhibition of 6-phosphogluconate dehydratase (GND) by phosphoenolpyruvate (PEP) was removed due to lack of experimental evidence. Inhibition of glucose-6-phosphate isomerase (PGI) by 6-phosphogluconate (PGN) [78] was added, and an error in the kinetic rate law of Entner-Doudoroff aldolase (EDA) in [32] was corrected. Reactions involved in acetate metabolism (acetyl-CoA synthetase, ACS; phosphotransacetylase, PTA; acetate kinase, ACK) were taken from [31]. The rate law of ACS was modified to be function of the concentration of the cofactors that are actually involved in this reaction (ATP and CoA) instead of NADP.

#### Oxidative phosphorylation

In aerobic conditions, *E*. *coli* uses oxidative phosphorylation to produce ATP. The main components of this pathway are two NADH dehydrogenases (reactions NDHI and NDHII), the succinate dehydrogenase complex (reactions SDH and SQR), the cytochrome *bo* oxidase (CYTBO) and the ATP synthase (ATP_SYN) [79]. NDHI and NDHII catalyze the transfer of electrons from NADH to the quinone pool (Q) in the cytoplasmic membrane. In contrast to NDHII, NDHI also generates a proton gradient by translocating H^+^ from cytoplasm to periplasm, with an H^+^/e^-^ ratio of 2 [79]. SQR is a complex of four proteins (SdhA, B, C and D). SdhA is a part of the TCA cycle (reaction SDH) and oxidizes succinate to fumarate by reducing FAD to FADH_2_. Further transfer of electrons from FADH_2_ to Q (reaction SQR) occurs via the three other proteins. CYTBO couples the two-electron oxidation of ubiquinol (QH_2_) with the four-electron reduction of molecular oxygen to water. This enzyme also functions as a proton pump, with an H^+^/e^-^ ratio of 2 [79]. The proton gradient is eventually used by the ATP synthase to generate ATP, with an H^+^/ATP ratio of 4 [80]. Oxidative phosphorylation was modelled using mass action kinetics. The sigmoidal dependence of the rates of H^+^ pumps (NDHI and CYTBO) and ATP synthase on pH gradient across the membrane was considered using the relation proposed by [81]. The rate of these reactions was modeled using the following rate laws:

$$v_{NDHI}=V_{max}.\frac{1}{1+\Delta pH^{2}}.\left(NADH.Q-\frac{NAD.QH_{2}}{K_{eq}}\right)$$ (1)

$$v_{NDH2}=V_{max}.\left(NADH.Q-\frac{NAD.QH_{2}}{K_{eq}}\right)$$ (2)

$$v_{SQR}=V_{max}.\left(FADH_{2}.Q-\frac{FAD.QH_{2}}{K_{eq}}\right)$$ (3)

$$v_{CYTBO}=V_{max}.\frac{1}{1+\Delta pH^{2}}.\left(QH_{2}².O_{2}-\frac{Q²}{K_{eq}}\right)$$ (4)

$$v_{ATP\_SYN}=V_{max}.\frac{\Delta pH^{4}}{1+\Delta pH^{4}}.\left(ADP.P-\frac{ATP}{K_{eq}}\right)$$ (5)

with

$$\Delta pH=\text{log}\left(\frac{H_{cytoplasm}}{H_{periplasm}}\right)$$ (6)

#### Interconversion of nucleotides and reduced cofactors

Several processes strongly impact the balance of ATP/ADP/AMP/cAMP pools and had to be considered to fit the experimental data: *i*) non-growth associated processes which consume ATP are lumped in the reaction ATP_NGAM, *ii*) adenylate kinase (reaction ADK), which catalyzes the reversible conversion of AMP and ATP into two molecules of ADP, *iii*) adenylate cyclase (CYA), which catalyzes the synthesis of cAMP from ATP, and *iv*) cAMP phosphodiesterase (DOS), which hydrolyses cAMP into AMP. The reversible reduction of NADP by NADH is catalyzed by two transhydrogenases [56] lumped into the reaction PNT. These reactions were modeled using mass action kinetics.

#### Growth

An overall pseudo-reaction was defined to describe cellular growth in terms of all the required metabolic precursors, with stoichiometric coefficients taken from the biomass function of iAF1260 [36]. Assuming the growth rate (μ) is controlled by intracellular concentration of all the cell building blocks *S*_*i*_, the rate of this reaction was modeled using the following equation:

$$\mu =V_{max}.\prod_{i}\frac{S_{i}}{S_{i}+K_{m}^{S_{i}}}$$ (7)

### Parameter estimation

Values of 56% of the parameters (253 on a total of 449) were directly taken from the literature. Parameters not available in the literature, which do not have a real biochemical estimate (*e*.*g*. Michaelis constants of the biomass function), or for which biochemical measurements are generally not representative of intracellular conditions (*e*.*g*. *Vmax*) were estimated to reproduce in the best possible way 276 experimental data obtained from *E*. *coli* K-12 MG1655 grown on glucose, under aerobic condition, at a dilution rate of 0.1 h^-1^. These data were steady state fluxes and metabolite concentrations [13, 34, 36, 37, 82] and time-course concentrations of intracellular metabolites in response to a glucose pulse [35] (S1 Dataset). Parameter estimation was formulated as a constrained optimization problem:

minimize f(p)

subject to g(p)≥c

where *p* is the parameter vector, *f* is the objective function which evaluates the deviation between the simulated and measured data, *g(p)* is the constraint function, and *c* is the constraint vector. The objective function *f* was defined as the sum of squared weighted errors:

$$f\left(p\right)=\underset{i}{\sum }{\left(\frac{x_{i}-y_{i}\left(p\right)}{\sigma_{i}}\right)}^{2}$$

where *x*_*i*_ is the experimental value of the data point *i*, with experimental standard deviation *σ*_*i*_, and *y*_*i*_*(p)* is the corresponding simulated value. Constraints were defined on estimated parameters (10^−4^ mM ≤ *K*_*M*_ ≤ 10^3^ mM; 10^−2^ mM/s ≤ *Vmax* ≤ 10^3^ mM/s; 10^−4^ ≤ *K*_*eq*_ ≤ 10^6^) to ensure they are kept within a biologically reasonable range. The objective function was minimized with the Particle Swarm Optimization algorithm [83], using the Condor-COPASI system [84] on a pool of 2500 CPU cores. The experimental and fitted data are provided in Supporting Information (S1 Dataset). Values of all the parameters (and the corresponding references for those values taken from the literature) are given in Supporting Information (S1 Text).

### Model analysis and validation

Analyses described below were performed using R (v3.0, www.r-project.org) after converting the model into Fortran. All the scripts are provided in Supporting Information (S1 Code).

#### Simulations

Steady-states were calculated using the *runsteady* function of the *rootSolve* R package (v1.6.5). Absolute and relative error tolerances were set to 10^−8^ and 10^−6^, respectively.

#### Metabolic control analysis

Scaled flux ($C_{E}^{J}$) and concentration ($C_{E}^{M}$) controls, which represent the fractional change in the steady-state flux *J* and metabolite *M* in response to a fractional change in the rate of the step *E* coefficients, were calculated as follows:

$$C_{E}^{J}=\frac{\partial lnJ}{\partial lnE}$$ (8)

and

$$C_{E}^{M}=\frac{\partial lnM}{\partial lnE}$$ (9)

The overall flux (*CJ_E_*) and concentration (*CC_E_*) controls exerted on the system by the step *E* were calculated as the L2-norm of all its control coefficients:

$$CJ_{E}=\sqrt{\sum_{J}{\left(C_{E}^{J}\right)}^{2}}$$ (10)

and

$$CC_{E}=\sqrt{\sum_{M}{\left(C_{E}^{M}\right)}^{2}}$$ (11)

To evaluate the sensitivity of these coefficients to parameters, this analysis was performed for 100,000 sets of random parameters uniformly sampled within ±20% around their reference values.

#### Sampling of the solution space

A total of 600,000 flux distributions were uniformly sampled within the solution space of a stoichiometric version of the kinetic model, using the Cobra Toolbox (v2.0.5) and Matlab 2013. In parallel, the *runif* function of R was used to generate 600,000 random sets of enzyme levels, and the corresponding steady-states were simulated under excess glucose (concentration fixed at 10 mM). For each set, enzyme levels were sampled over two orders of magnitude (between 0.1 and 10 times the *Vmax* of the initial model) from a log uniform distribution, which ensures each order of magnitude to be sampled in similar proportions.

#### Identification of functional couplings

For each steady state, the following variables were calculated: growth rate, glucose and oxygen uptake rates, ATP, NADH and NADPH production rates and yields, sum of fluxes, cost of enzymes, biomass yield, total intracellular metabolites pool, ATP production rate and enzyme cost per sum of fluxes, and sum of fluxes per glucose consumed. Enzyme cost, defined as the total amount of amino acids present in central metabolic enzymes, was calculated by summing for each reaction the product of enzyme concentration by the number of amino acids in the corresponding enzyme, similarly to [85, 86]. Enzyme concentrations were taken from [63], those not available were assumed to be the average concentration of other enzymes (18 μM). Pairwise relationships between steady-state systemic variables and (absolute and relative) intracellular fluxes through the main pathways were evaluated using Spearman correlation test (*cor* function of R *stats* package) and mutual information (*mutinformation* function of *infotheo* R package v1.2.0).

#### Model validation

Exploration of the model solution space suggested the existence of robust couplings between several metabolic processes (carbon uptake, catabolism, energy and redox production, and growth). We independently validated these model predictions based on a test set of experimentally observed phenotypes that were not used during model construction. We collected from the literature a total of 778 intracellular and extracellular flux data from some 266 experiments where several wild-type and mutant *E*. *coli* K-12 strains (MG1655 and its close derivatives JM101, BW25113, W3110 and TG1) were grown in different conditions (deep well plate, bioreactor, shake flask, and chemostat) (S2 Dataset) [5, 27, 34, 53–67]. This data set therefore represents a very broad range of the metabolic states that can be expressed by *E*. *coli* growing on glucose. The testing data set is very different from the training data set, which contains experimental data from a single wild-type *E*. *coli* strain grown in a unique condition. This provides a robust assessment of the predictive ability of the model.

The good agreement between simulated and experimental validation data (Figs 4–6) indicates the model yielded fairly accurate predictions of the metabolic states that can be expressed by *E*. *coli* growing on glucose. Glucose uptake, catabolism, energy and redox production and growth were predicted to be strongly coupled despite large, random changes of gene expression. All the experimental data support the model-driven hypothesis that metabolic regulation is sufficient to maintain the tight coordination between these key metabolic processes.

## Supporting information

S1 Table Comparison of available detailed kinetic models of *Escherichia coli* metabolism. (XLSX)

S1 Text Extended information on the kinetic model of *Escherichia coli* metabolism. (PDF)

S1 Model Kinetic model of *Escherichia coli* metabolism. (ZIP)

S1 Dataset Experimental data used to calibrate the model. (XLSX)

S2 Dataset Experimental data used to validate the model predictions. (XLSX)

S1 Code R and Matlab scripts used for model analysis. (ZIP)
