# Supplementary material for: Metabolic regulation is sufficient for global and robust coordination of glucose uptake, catabolism, energy production and growth in Escherichia coli
Source: PLoS Comput Biol. 2017 Feb 10;13(2):e1005396. doi: 10.1371/journal.pcbi.1005396 (PMC5328398; doi:10.1371/journal.pcbi.1005396)
Supplement: S1 Text — (PDF) [file pcbi.1005396.s002.pdf]

Kinetic model of *Escherichia coli* central metabolism

-

Documentation

Pierre Millard[1,2,3], Kieran Smallbone[1,2] and Pedro Mendes[1,2,4]

[1]MCISB, Manchester Institute of Biotechnology, University of Manchester, M17DN, Manchester, UK.

[2]School of Computer Science, University of Manchester, Manchester, UK.

[3]LISBP, Université de Toulouse, CNRS, INRA, INSA, Toulouse, France.

[4]Center for Quantitative Medicine and Dept. Cell Biology, UConn Health, Farmington CT 06030, USA.

Contents

1. **Model overview** 3
2. **Model units** 4
3. **Reactions** 4
4. **ODEs system** 9
5. **Rate laws** 11
6. **Conservation laws** 16
7. **Concentrations of cofactors and conserved moieties** 16
8. **Magnesium complexes** 17
9. **Model calibration** 17
10. **Model validation** 26
11. **References** 27

1. Model overview

The model developed in this work represents the central metabolic network of the bacterium *Escherichia coli*. It should be noted that when developing a model, certain criteria must be decided, such as the level of detail and the boundaries within which the model can be expected to be valid. The current model simulates the metabolic operation of *E. coli* K-12 MG1655 during exponential growth phase, under aerobic condition and glucose limitation ($\mu = 0.1\ h^{-1}$). It may allow simulation of other scenarios by changing the enzyme activities to reflect the altered conditions, and/or implementing additional pathways known to be active in the other scenarios.

This model comprises three compartments: the environment and the cell which is divided in two compartments (cytoplasm and periplasm). The periplasmic volume represents 20% of the cell volume [1]. The model contains 77 species and 68 reactions constitutive of the central carbon and energy pathways of *E. coli* (Figure 1):

- transport reactions between the environment and the periplasm
- glucose phosphotransferase system (PTS)
- glycolytic and gluconeogenic pathways (EMP)
- pentose phosphate pathway (PP)
- Entner-Doudoroff pathway (ED)
- anaplerotic reactions (AR)
- tricarboxylic acids cycle (TCA)
- glyoxylate shunt (GS)
- acetate metabolism
- oxidative phosphorylation (OP)
- synthesis of biomass

The following sections describe:

- all the reactions included in the model
- the system of ODEs
- the laws for conserved moieties
- the rate laws for each reaction
- the value of all kinetic parameters

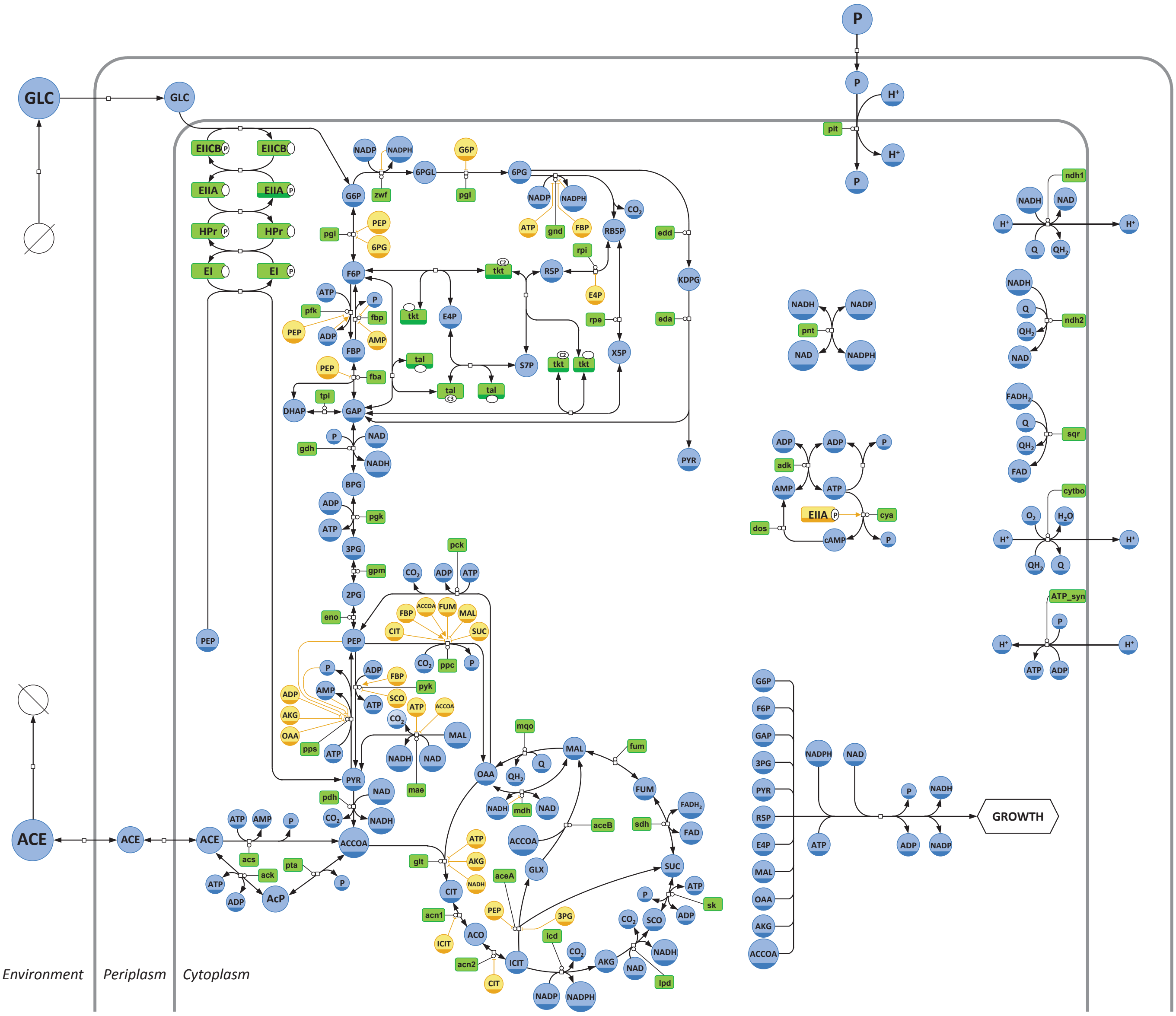

Figure 1. Central metabolic network of *E. coli* implemented in the model. The model comprises three compartments: the environment, the periplasm and the cytoplasm. Metabolites are shown in blue (reactants) and orange (regulators). Enzymes are shown in green. Black and orange arrows denote reactions and regulatory interactions, respectively. The diagram adopts the conventions of the Systems Biology Graphical Notation process description [2].

2. Model units

Model units are millimole (mmol) for amounts, litre (L) for volumes, and second (s) for time. Experimental data used for parameter estimation were converted into intracellular units (mM for concentrations and mM/s for fluxes) assuming a cytosolic volume of 1.77×10^{-3} L/g_{DW} [3].

3. Reactions

Table S1 lists the reactions implemented in the model. It also includes the types of kinetic equations describing each reaction and the effectors considered. When the equation was taken or adapted from the literature, the corresponding reference is given. All the equations can be found in section 5. This model is available in SBML and COPASI formats in Supplementary data and can be downloaded from the Biomodels database (http://www.ebi.ac.uk/biomodels/) with identifier < MODEL1515110000>.

Table S1. Reactions implemented in the model. When equations were taken from the literature, references are given in the *Rate law* column.

The following abbreviations are used: MA: Mass action; MM: Michaelis-Menten; MWC: Monod-Wyman-Changeux; OBB: Ordered Bi Bi; OUB: Ordered Uni Bi; PPTB: Ping Pong Ter Bi; PPUBBU: Ping Pong Uni Bi Bi Uni; RBB: Random Bi Bi; RBT: Random Bi Ter; RUB: Random Uni Bi. The signs (+) and (-) denotes a positive and a negative control of reaction rates by their effectors, respectively.

Sub-system	*Reaction name*	*EC number*	*Reaction*	*Effector(s)*	*Rate law*	*Comment*
-	GLC_feed	-	∅ → GLC_{env}	-	Constant flux	Glucose inflow into the environment
-	ACE_OUT	-	ACE_{env} → ∅	-	MA	Acetate output from the environment
Exchange reactions	XCH_GLC	-	GLC_{env} ↔ GLC_{per}	-	MM	Additional information are given after the table
	XCH_P	-	P_{env} ↔ P_{per}	-	MM	
	XCH_ACE	-	ACE_{per} ↔ ACE_{env}	-	MM	
Glucose uptake	PTS_0	2.7.3.9	ei + PEP ↔ eiP + PYR	-	MA [4]	-
	PTS_1	2.7.1.199	hpr + eiP ↔ hprP + ei	-	MA [4]	-
	PTS_2	2.7.1.199	eiia + hprP ↔ eiiaP + hpr	-	MA [4]	-
	PTS_3	2.7.1.199	eiicb + eiiaP ↔ eiicbP + eiia	-	MA [4]	-
	PTS_4	2.7.1.199	GLC_{per} + eiicbP ↔ G6P + eiicb	-	MA [4]	-
Phosphate uptake	PIT	-	P_{per} + H^{+}_{per} ↔ P_{cyt} + H^{+}_{cyt}	H^{+}_{per} (+)	MM	-
Glycolysis & gluconeogenesis	PGI	5.3.1.9	G6P ↔ F6P	PEP (-), PGN (-)	MM, adapted from [5]	With inhibition by PGN [6, 7]
	PFK	2.7.1.11	ATP + F6P ↔ ADP + FDP	PEP (-)	MWC [5]	-
	FBP	3.1.3.11	FDP → F6P + P	AMP (-), P (-)	MWC [5]	-
	FBA	4.1.2.13	FDP ↔ DAP + GAP	PEP (-)	OUB [5]	-
	TPI	5.3.1.1	DAP ↔ GAP	-	MM [5]	-
	GDH	1.2.1.12	GAP + NAD + P ↔ BPG + NADH	-	RBT [5]	-
	PGK	2.7.2.3	ADP + BPG ↔ ATP + PGA3	-	RBB [5]	-
	GPM	5.4.2.11/12	PGA3 ↔ PGA2	-	MM [5]	-
	ENO	4.2.1.11	PGA2 ↔ PEP	-	MM [5]	-
	PYK	2.7.1.40	ADP + PEP → ATP + PYR	SUCCOA (-), FDP (+)	MWC [5]	-
	PPS	2.7.9.2	ATP + PYR ↔ AMP + PEP + P	ADP (-), AKG (-), OAA (-), AMP (-), P (-), PEP (-)	PPUBBU [5]	-
	PDH	1.2.1.-	COA + NAD + PYR ↔ ACCOA + NADH + HCO^{3-}	-	PPTB [5]	-
PP pathway	ZWF	1.1.1.49	G6P + NADP ↔ GL6P + NADPH	NADPH (-)	RBT [5]	-
	PGL	3.1.1.31	GL6P ↔ PGN	G6P (-)	MM [5]	-
	GL6P_HYDRO	-	GL6P ↔ PGN	-	MA [5]	Spontaneous hydrolysis of GL6P
	GND	1.1.1.44	NADP + PGN ↔ NADPH + RU5P + HCO^{3-}	FDP (-), ATP (-), NADPH (-)	RBT, adapted from [5]	Inhibition by PEP was removed (no experimental evidence)
	RPE	5.1.3.1	RU5P ↔ X5P	-	MM [5]	-
	RPI	5.3.1.6	RU5P ↔ R5P	E4P (-)	MM [5]	-
	X5P_GAP_TKT	2.2.1.1	tkt + X5P ↔ GAP + tktC2	-	MA [5]	-
	F6P_E4P_TKT	2.2.1.1	E4P + tktC2 ↔ F6P + tkt	-	MA [5]	-
	S7P_R5P_TKT	2.2.1.1	R5P + tktC2 ↔ S7P + tkt	-	MA [5]	-
	F6P_GAP_TAL	2.2.1.2	GAP + talC3 ↔ F6P + tal	-	MA [5]	-
	S7P_E4P_TAL	2.2.1.2	S7P + tal ↔ E4P + talC3	-	MA [5]	-

ED pathway	EDD	4.2.1.12	PGN ↔ KDPG	-	MM [5]	-
	EDA	4.1.2.14	KDPG ↔ GAP + PYR	-	OUB [5]	-
Anaplerotic reactions	PPC	4.1.1.31	PEP + HCO^{3-} ↔ OAA + P	ACCOA (+), CIT (-), FDP (+), FUM (-), MAL (-), SUC (-), ASP (+), CYS (-)	MWC [5]	-
	PCK	4.1.1.49	ATP + OAA ↔ ADP + PEP + HCO^{3-}	-	RBT [5]	-
	MAD	1.1.1.39	MAL + NAD → NADH + PYR + HCO^{3-}	ATP (-), ACCOA (-), COA (-), ASP (+)	MWC [5]	-
TCA cycle	GLT	2.3.3.1	ACCOA + OAA ↔ CIT + COA	ATP (-), AKG (-), NADH (-)	MWC [5]	-
	ACN_1	4.2.1.3	CIT ↔ ACO	ICIT (-)	MM [5]	-
	ACN_2	4.2.1.3	ACO ↔ ICIT	CIT (-)	MM [5]	-
	ICD	1.1.1.42	ICIT + NADP ↔ AKG + NADPH + HCO^{3-}	-	MWC [5]	-
	ACEK_1	3.1.3.-	ATP + icd ↔ ADP + icdP	-	MA [5]	-
	ACEK_2	3.1.3.-	icdP ↔ icd + P	-	MA [5]	-
	LPD	1.2.4.2	COA + AKG + NAD → NADH + SUCCOA + HCO^{3-}	-	MWC [5]	-
	SK	6.2.1.5	ADP + SUCCOA + P ↔ ATP + COA + SUC	-	RBT [5]	-
	SDH	1.3.5.1	Q + SUC ↔ FUM + QH_2	-	RBB [5]	-
	FUMA	4.2.1.2	FUM ↔ MAL	-	MM [5]	-
	MQO	1.1.5.4	MAL + Q ↔ OAA + QH_2	-	MM [5]	-
	MDH	1.1.1.37	QH_2 + OAA ↔ MAL + Q	-	OBB [5]	-
Glyoxylate shunt	ACEA	4.1.3.1	ICIT ↔ GLX + SUC	PEP (-), PGA3 (-)	RUB [5]	-
	ACEB	2.3.3.9	ACCOA + GLX ↔ COA + MAL	-	RBB [5]	-
Acetate metabolism	PTA	2.3.1.8	ACCOA + P ↔ COA + ACP	-	MM [8]	-
	ACK	2.7.2.1	ACP + ADP ↔ ACE_{per} + ATP	-	MM [8]	-
	ACS	6.2.1.1	ACE_{per} + ATP + COA → ACCOA + AMP + 2 * P	-	MM [8]	-
Oxidative phosphorylation	NDHI	1.6.5.3	NADH + Q + 4 * H^+_{cyt} ↔ NAD + QH_2 + 4 * H^+_{per}	H^+_{per} (-)	MA	Additional information are given after the table
	NDHII	1.6.5.9	NADH + Q ↔ NAD + QH_2	-	MA	
	SQR	1.3.5.1	$FADH_2$ + Q ↔ FAD + QH_2	-	MA	
	CYTBO	1.10.3.10	2 * QH_2 + 8 * H^+_{cyt} + O_2 ↔ 2 * Q + 8 * H^+_{per} + 2 * H_2O	H^+_{per} (-)	MA	
	ATP_SYN	3.6.3.14	ADP + P + 4 * H^+_{per} ↔ ATP + 4 * H^+_{cyt}	H^+_{per} (+)	MA	
Additional reactions for nucleotides and redox cofactors	PNT	1.6.1.1/2/3	NAD + NADPH ↔ NADH + NADP	-	MA	Additional information are given after the table
	ADK	2.7.4.3	AMP + ATP ↔ 2 * ADP	-	MA	
	ATP_NGAM	-	ATP ↔ ADP + P	-	MA	
	CYA	4.6.1.1	ATP ↔ cAMP + 2 * P	eiiaP (+)	MA	
	DOS	3.1.4.53	cAMP ↔ AMP	-	MA	
Biomass synthesis	GROWTH	-	116 * G6P + 204 * E4P + 845 * PGA3 + 1010 * OAA + 610 * AKG + 1601 * PYR + 507 * R5P + 293 * PEP + 73 * GAP + 40 * F6P + 10169 * NADPH + 2118 * ACCOA + 2004 * NAD + 30508 * ATP → 10169 * NADP + 2118 * COA + 2004 * NADH + 30508 * ADP + 30508 * P	-	Random ordered	Additional information are given after the table

All the rate laws are given in section 5. Details on the equations taken from the literature can be found in the original paper given in reference. Information on the modelling of some processes is detailed hereafter.

- *Exchange reactions*

Three reactions enable the transport of glucose (XCH_GLC), acetate (XCH_ACE) and phosphate (XCH_P) between the environment and the periplasm. Diffusion through the outer membrane is modelled as a saturable, porin-facilitated diffusion process [9], using reversible Michaelis-Menten kinetics. The same (arbitrary) values for V_{max} and K_m of 100 mM/s and 10 mM were taken for all compounds.

- *Oxidative phosphorylation*

Oxidative phosphorylation is used by *E. coli* to generate ATP. In aerobic condition, electrons are transferred from NADH and $FADH_2$ to O_2. This process generates an H^+ gradient across the cytoplasmic membrane, which is then used to drive ATP synthesis. The main components of oxidative phosphorylation are two NADH dehydrogenases (NDHI and NDHII), the succinate dehydrogenase complex (SQR), the cytochrome bo oxidase (CYTBO) and the ATP synthase (ATP_SYN) [10]. *E. coli* also has two other cytochromes (bd1 and bd2) which are expressed under oxygen-limited condition and starvation for carbon and/or phosphate [11-13], respectively, and were not included in the model since they are not significantly active in exponential growth on glucose in aerobic condition. NDHI and NDHII catalyse the transfer of electrons from NADH to the quinone pool (Q) in the cytoplasmic membrane. In contrast to NDHII, NDHI also generates a proton gradient by translocating H^+ from cytoplasm to periplasm, with an H^+/e^- ratio of 2 [10]. SQR is a complex of 4 proteins (SDHA, B, C and D). SDHA is a part of the TCA cycle (reaction SDH) and oxidizes succinate to fumarate by reducing FAD to $FADH_2$. Further transfer of electrons from $FADH_2$ to Q (reaction SQR) occurs via the three other proteins. CYTBO couples the two-electron oxidation of ubiquinol (QH_2) with the four-electron reduction of molecular oxygen to water. It also functions as a proton pump, with an H^+/e^- ratio of 2 [10]. Finally, the proton gradient is used by the ATP synthase to generate ATP by translocating H^+ from the periplasm to the cytoplasm, with an H^+/ATP ratio of 4 [14].

Oxidative phosphorylation was modelled using reversible mass action kinetics. Although the oxidative phosphorylation reactions have no specific feedback regulation, experimental evidences show that kinetics of H^+ pumps (NDHI and CYTBO) and ATP_SYN strongly depends on the H^+ gradient. As the H^+ gradient increases, the reaction rate through NDHI and CYTBO decreases in a sigmoidal fashion. In opposite, a strongly sigmoidal increase of the rate of ATP synthesis with the increase of H^+ gradient was observed. We considered the dependence of reaction rates on the H^+ gradient using the relation proposed by [15].

E. coli maintains a cytoplasmic pH within a narrow range, approximately 7.4 to 7.8, when grown over a large range of environmental pH from pH 5 to 9 [16-18]. Thus, the cytosolic concentration of H^+ ions was fixed at 3.16×10^{-5} mM (pH=7.5, [18]).

- *Transport of phosphate*

Phosphate enters in the cytoplasm via the PIT transporter. Transport is energised by the proton gradient with a H^+/P ratio of 1 and can be abolished with uncouplers or respiration inhibitors, thus the reaction rate was modelled as function of the H^+ gradient, similarly to reactions of oxidative phosphorylation involved in the production of the H^+ gradient.

- *Additional reactions for nucleotides and redox cofactors*

Various processes strongly impact the balance of AMP, ADP, ATP and cAMP pools and had to be considered to fit the experimental data. Several reactions of non-growth associated processes which consume ATP were lumped in the reaction ATP_NGAM. Adenylate kinase (reaction ADK) catalyzes the reversible conversion of AMP and ATP to two molecules of ADP. Adenylate cyclase (CYA) catalyzes the synthesis of cAMP from ATP and is activated by the phosphorylated form of EIIA enzyme of the PTS. Finally, cAMP can be hydrolyzed into AMP by the cAMP phosphodiesterase (DOS).

The reversible reduction of NADP by NADH is catalysed by two transhydrogenases [19] lumped into the reaction PNT and modelled using reversible mass action kinetics.

- *Biomass synthesis*

In contrast to previous models where growth was function of extracellular glucose levels, we assumed that the growth rate is controlled by the intracellular concentration of the cell building blocks (G6P, E4P, PGA3, OAA, AKG, PYR, R5P, PEP, GAP, F6P, NADPH, ACCOA, NAD, ATP). Thus, we defined an overall pseudo-reaction to describe cellular growth in terms of the required metabolic precursors, with the stoichiometric coefficients taken from the biomass function published in [20] (after unit conversion from mmol/g_{DW}/h to mmol/$L_{cytoplasm}$/s). The kinetic equation for growth is:

$$\mu = V_{max} \cdot \prod_i \frac{S_i}{S_i + K_m^{S_i}}$$

where S_i represents the concentration of the building block i and $K_m^{S_i}$ represents the saturation of the growth rate with respect to the concentration of the metabolite i.

4. ODEs system

The differential equations, which describe the progression of the variables over time as a function of the system's rates, balance the:

- concentrations of extracellular metabolites (glucose, phosphate and acetate)
- concentrations of intracellular metabolites
- phosphorylation states of PTS proteins
- states of transaldolases and transketolases

<u>Metabolites:</u>

d(ACCOA)/dt = v_PDH - v_GLT - v_ACEB - 2118 * v_GROWTH + v_ACS - v_PTA

d(ACO)/dt = v_ACN_1 - v_ACN_2

d(ACE)/dt = v_ACK - v_ACS - v_XCH_ACE1

d(ACEp)/dt = (v_XCH_ACE1 - v_XCH_ACE2)* vol_cyt/vol_per

d(ACEx)/dt = v_ XCH_ACE2 * vol_per/vol_env - v_ACE_OUT

d(ACP)/dt = v_PTA - v_ACK

d(ADP)/dt = 2 * v_ADK - v_ATP_SYN - v_PGK + v_PFK - v_PYK + v_PCK - v_SK + v_ACEK_1 - v_ACK + 30508 * v_GROWTH

d(AKG)/dt = v_ICD - v_LPD - 610 * v_GROWTH

d(AMP)/dt = v_DOS - v_ADK + v_PPS + v_ACS

d(ATP)/dt = v_ATP_SYN - v_CYA - v_ADK + v_PGK - v_PFK + v_PYK - v_PCK - v_PPS + v_SK - v_ACEK_1 - v_ACS + v_ACK - 30508 * v_GROWTH

d(BPG)/dt = v_GDH - v_PGK

d(CAMP)/dt = v_CYA - v_DOS

d(CIT)/dt = v_GLT - v_ACN_1

d(DAP)/dt = v_FBA - v_TPI

d(E4P)/dt = v_S7P_E4P_TAL - v_F6P_E4P_TKT - 204 * v_GROWTH

d(F6P)/dt = v_PGI - v_PFK + v_F6P_E4P_TKT + v_F6P_GAP_TAL + v_FBP - 40 * v_GROWTH

d(FAD)/dt = v_SQR - v_SDH

d($FADH_2$)/dt = v_SDH - v_SQR

d(FDP)/dt = v_PFK - v_FBA - v_FBP

d(FUM)/dt = v_SDH - v_FUMA

d(G6P)/dt = v_PTS_4 - v_PGI - v_ZWF - 116 * v_GROWTH

d(GAP)/dt = v_FBA + v_TPI - v_GDH + v_X5P_GAP_TKT - v_F6P_GAP_TAL + v_EDA - 73 * v_GROWTH

d(GL6P)/dt = v_ZWF - v_PGL - v_GL6P_HYDRO

d(GLCp)/dt = (v_GLC_XCH - v_PTS_4)* vol_cyt /vol_per

d(GLCx)/dt = (v_GLC_feed - v_GLC_XCH)* vol_per/vol_env

d(GLX)/dt = v_ACEA - v_ACEB

d(Hp)/dt = (4 * v_NDHI + 8 * v_CYTBO - 4 * v_ATP_SYN - v_PIT) * vol_cyt/vol_per

d(ICIT)/dt = v_ACN_2 - v_ICD - v_ACEA

d(KDPG)/dt = v_EDD - v_EDA

d(MAL)/dt = v_FUMA - v_MAD + v_MDH - v_MQO + v_ACEB

d(NAD)/dt = v_MDH - v_PNT - v_GDH - v_MAD - v_PDH - v_LPD - 2004 * v_GROWTH

d(NADH)/dt = v_GDH + v_MAD + v_PDH + v_LPD - v_MDH + v_NADH_req + v_PNT - 2004 * v_GROWTH

d(NADP)/dt = v_PNT - v_GND - v_ZWF - v_ICD + 10169 * v_GROWTH

d(NADPH)/dt = v_GND + v_ZWF + v_ICD - v_PNT - 10169 * v_GROWTH

d(OAA)/dt = v_PPC - v_PCK - v_GLT + v_MQO - v_MDH - 1010 * v_GROWTH

d(Pp)/dt = (v_P_XCH - v_PIT)* vol_cyt/vol_per

d(Pc)/dt = v_FBP - v_GDH + v_PPC + v_PPS - v_SK + v_ACEK_2 - v_ATP_SYN + 2 * v_CYA + 2 * v_ACS - v_PTA + v_ATP_MAINTENANCE + 30508 * v_GROWTH + v_PIT

d(PEP)/dt = v_ENO - v_PYK - v_PPC + v_PCK + v_PPS - v_PTS_0 - 293 * v_GROWTH

d(PGA2)/dt = v_GPM - v_ENO

d(PGA3)/dt = v_PGK - v_GPM - 845 * v_GROWTH

d(PGN)/dt = v_PGL - v_GND - v_EDD

d(PYR)/dt = v_PYK - v_PPS + v_MAD - v_PDH + v_EDA + v_PTS_0 - 1601 * v_GROWTH

d(Q)/dt = v_MDH + 2 * v_CYTBO - v_SQR - v_NDHI - v_NDHII - v_MQO

d(QH_2)/dt = v_SQR + v_NDHI + v_NDHII + v_MQO - v_MDH - 2 * v_CYTBO

d(R5P)/dt = v_RPI - v_S7P_R5P_TKT - 507 * v_GROWTH

d(RU5P)/dt = v_GND - v_RPE - v_RPI

d(S7P)/dt = v_S7P_R5P_TKT - v_S7P_E4P_TAL

d(SUC)/dt = v_SK - v_SDH + v_ACEA

d(SUCCOA)/dt = v_LPD - v_SK

d(X5P)/dt = v_RPE - v_X5P_GAP_TKT

Proteins:

d(ei)/dt = v_PTS_1 - v_PTS_0

d(eiia)/dt = v_PTS_3 - v_PTS_2

d(eiiaP)/dt = v_PTS_2 - v_PTS_3

d(eiicb)/dt = v_PTS_4 - v_PTS_3

d(eiicbP)/dt = v_PTS_3 - v_PTS_4

d(eiP)/dt = v_PTS_0 - v_PTS_1

d(hpr)/dt = v_PTS_2 - v_PTS_1

d(hprP)/dt = v_PTS_1 - v_PTS_2

d(icd)/dt = v_ACEK_2 - v_ACEK_1

d(icdP)/dt = v_ACEK_1 - v_ACEK_2

d(tal)/dt = v_F6P_GAP_TAL - v_S7P_E4P_TAL

d(talC3)/dt = v_S7P_E4P_TAL - v_F6P_GAP_TAL

d(tkt)/dt = v_S7P_R5P_TKT - v_X5P_GAP_TKT + v_F6P_E4P_TKT

d(tktC2)/dt = v_X5P_GAP_TKT - v_F6P_E4P_TKT - v_S7P_R5P_TKT

5. Rate laws

This section contains the rate laws for each reaction.

$$vACEA = \frac{\frac{Vmax \cdot \left(ICIT - \frac{GLX \cdot SUC}{Keq}\right)}{KmICIT}}{1 + \frac{ICIT}{KmICIT} \cdot \left(1 + \frac{PEP}{KdPEPicit}\right) + \frac{SUC}{KdSUC} \cdot \left(1 + \frac{ICIT}{KdICITsuc}\right) + \frac{KmSUC}{KdSUC} \cdot \frac{GLX}{KmGLX} \cdot \left(1 + \frac{PEP}{KdPEPglx}\right) + \frac{GLX}{KmGLX} \cdot \frac{SUC}{KdSUC} + \frac{PEP}{KdPEP} + \frac{PGA3}{KdPGA3}}$$

$$vACEB = \frac{\frac{Vmax \cdot \left(ACCOA \cdot GLX - \frac{COA \cdot MAL}{Keq}\right)}{KmACCOA \cdot KmGLX}}{\left(1 + \frac{ACCOA}{KmACCOA}\right) \cdot \left(1 + \frac{GLX}{KmGLX}\right) + \left(1 + \frac{COA}{KmCOA}\right) \cdot \left(1 + \frac{MAL}{KmMAL}\right) - 1}$$

$$vACEK\_1 = k \cdot \left(ATP \cdot icd - \frac{ADP \cdot icdP}{Keq}\right)$$

$$vACEK\_2 = k \cdot \left(icdP - \frac{icd \cdot P}{Keq}\right)$$

$$vACK = \frac{\frac{Vmax \cdot \left(ACP \cdot ADP - \frac{ACEx \cdot ATP}{Keq}\right)}{KmACP \cdot KmADP}}{\left(1 + \frac{ACP}{KmACP} + \frac{ACEx}{KmACE}\right) \cdot \left(1 + \frac{ADP}{KmADP} + \frac{ATP}{KmATP}\right)}$$

$$vACE\_OUT = D \cdot ACEx$$

$$vACN1 = \frac{\frac{Vmax \cdot \left(CIT - \frac{ACO}{Keq}\right)}{KmCIT}}{1 + \frac{CIT}{KmCIT} + \frac{ACO}{KmACO} + \frac{ICIT}{KmICIT}}$$

$$vACN2 = \frac{\frac{Vmax \cdot \left(ACO - \frac{ICIT}{Keq}\right)}{KmACO}}{1 + \frac{ACO}{KmACO} + \frac{ICIT}{KmICIT} + \frac{CIT}{KmCIT}}$$

$$vACS = \frac{\frac{Vmax \cdot ACEx \cdot ATP \cdot COA}{KmACE \cdot KmATP \cdot KmCOA}}{\left(1 + \frac{ACEx}{KmACE}\right) \cdot \left(1 + \frac{ATP}{KmATP}\right) \cdot \left(1 + \frac{COA}{KmCOA}\right)}$$

$$vADK = k \cdot \left(AMP \cdot ATP - \frac{ADP^2}{Keq}\right)$$

$$vATP\_NGAM = Vmax \cdot \left(ATP - \frac{ADP \cdot P}{Keq}\right)$$

$$vATP\_SYN = \frac{Vmax \cdot \left(\frac{ln\left(\frac{Hout}{Hin}\right)}{ln10}\right)^4}{1 + \left(\frac{ln\left(\frac{Hout}{Hin}\right)}{ln10}\right)^4} \cdot \left(ADP \cdot P - \frac{ATP}{Keq}\right)$$

$$vCYA = \frac{k \cdot \left(ATP - \frac{CAMP \cdot P^2}{Keq}\right) \cdot eiiaP}{eiiaP + KaeiiaP}$$

$$vCYTBO = \frac{Vmax}{1 + \left(\frac{ln\left(\frac{Hout}{Hin}\right)}{ln10}\right)^2} \cdot \left(QH2^2 \cdot O2 - \frac{Q^2}{Keq}\right)$$

$$vDOS = k \cdot \left(CAMP - \frac{AMP}{Keq} \right)$$

$$vEDA = \frac{\frac{Vmax \cdot \left(KDPG - \frac{GAP \cdot PYR}{Keq} \right)}{KmKDPG}}{1 + \frac{KDPG}{KmKDPG} + \left(1 + \frac{GAP}{KmGAP} \right) \cdot \left(1 + \frac{PYR}{KmPYR} \right) - 1}$$

$$vEDD = \frac{\frac{Vmax \cdot \left(PGN - \frac{KDPG}{Keq} \right)}{KmPGN}}{1 + \frac{PGN}{KmPGN} + \frac{KDPG}{KmKDPG}}$$

$$vENO = \frac{\frac{Vmax \cdot \left(PGA2 - \frac{PEP}{Keq} \right)}{KmPGA2}}{1 + \frac{PGA2}{KmPGA2} + \frac{PEP}{KmPEP}}$$

$$vF6P\_E4P\_TKT = kcat \cdot \left(E4P \cdot tktC2 - \frac{F6P \cdot tkt}{Keq} \right)$$

$$vF6P\_GAP\_TAL = kcat \cdot \left(GAP \cdot talC3 - \frac{F6P \cdot tal}{Keq} \right)$$

$$vFBA = \frac{\frac{Vmax \cdot \left(FDP - \frac{DAP \cdot GAP}{Keq} \right)}{KmFDP}}{1 + \frac{FDP}{KmFDP} + \frac{DAP}{KmDAP} + \frac{DAP}{KmDAP} \cdot \frac{GAP}{KmGAP} + \frac{PEP}{KmPEP}}$$

$$vFBP = \frac{\frac{\frac{Vmax \cdot n \cdot MgFDP}{KirFDPMg}}{\left(1 + \frac{KmrFDP}{KirFDP} \cdot \frac{MG}{KmrMg} + \frac{P}{KirP} + \frac{P}{KirP} \cdot \frac{MG}{KirPMg} + \frac{F6P}{KirF6P} + \frac{F6P}{KirF6P} \cdot \frac{MG}{KirF6PMg} + \frac{P}{KirP} \cdot \frac{F6P}{KirPF6P} + \frac{P}{KirP} \cdot \frac{F6P}{KirPF6P} \cdot \frac{MG}{KirPF6PMg} + \frac{FDP - MgFDP}{KirFDP} + \frac{KdFDPMg}{KmrMg} \cdot \frac{MgFDP}{KirFDP} + \frac{AMP}{KirAMP} + \frac{MgFDP}{KirFDPMg} + \frac{MgFDP}{KirFDPMg} \cdot \frac{MG}{KirFDPMgMg} + \frac{AMP}{KirAMP} \cdot \frac{FDP - MgFDP}{KirAMPFDP} \right)}}{1 + L0 \cdot \left(\frac{\left(1 + \frac{KmtFDP}{KitFDP} \cdot \frac{MG}{KmtMg} + \frac{P}{KitP} + \frac{P}{KitP} \cdot \frac{MG}{KitPMg} + \frac{F6P}{KitF6P} + \frac{F6P}{KitF6P} \cdot \frac{MG}{KitF6PMg} + \frac{P}{KitP} \cdot \frac{F6P}{KitPF6P} + \frac{P}{KitP} \cdot \frac{F6P}{KitPF6P} \cdot \frac{MG}{KitPF6PMg} + \frac{FDP - MgFDP}{KitFDP} + \frac{KdFDPMg}{KmtMg} \cdot \frac{MgFDP}{KitFDP} + \frac{AMP}{KitAMP} + \frac{MgFDP}{KitFDPMg} + \frac{MgFDP}{KitFDPMg} \cdot \frac{MG}{KitFDPMgMg} + \frac{AMP}{KitAMP} \cdot \frac{FDP - MgFDP}{KitAMPFDP} \right)}{\left(1 + \frac{KmrFDP}{KirFDP} \cdot \frac{MG}{KmrMg} + \frac{P}{KirP} + \frac{P}{KirP} \cdot \frac{MG}{KirPMg} + \frac{F6P}{KirF6P} + \frac{F6P}{KirF6P} \cdot \frac{MG}{KirF6PMg} + \frac{P}{KirP} \cdot \frac{F6P}{KirPF6P} + \frac{P}{KirP} \cdot \frac{F6P}{KirPF6P} \cdot \frac{MG}{KirPF6PMg} + \frac{FDP - MgFDP}{KirFDP} + \frac{KdFDPMg}{KmrMg} \cdot \frac{MgFDP}{KirFDP} + \frac{AMP}{KirAMP} + \frac{MgFDP}{KirFDPMg} + \frac{MgFDP}{KirFDPMg} \cdot \frac{MG}{KirFDPMgMg} + \frac{AMP}{KirAMP} \cdot \frac{FDP - MgFDP}{KirAMPFDP} \right)} \right)^{n}}$$

$$vFUMA = \frac{\frac{Vmax \cdot \left(FUM - \frac{MAL}{Keq} \right)}{KmFUM}}{1 + \frac{FUM}{KmFUM} + \frac{MAL}{KmMAL}}$$

$$vGDH = \frac{\frac{Vmax \cdot \left(P \cdot GAP \cdot NAD - \frac{BPG \cdot NADH}{Keq} \right)}{KmP \cdot KmGAP \cdot KmNAD}}{\left(1 + \frac{P}{KmP} \right) \cdot \left(1 + \frac{GAP}{KmGAP} \right) \cdot \left(1 + \frac{NAD}{KmNAD} \right) + \left(1 + \frac{BPG}{KmBPG} \right) \cdot \left(1 + \frac{NADH}{KmNADH} \right) - 1}$$

$$vGL6P\_HYDROLYSIS = KGl6Phydrol \cdot \left(GL6P - \frac{PGN}{KeqGl6Phydrol} \right)$$

$$vGLT = \frac{\frac{Vmax \cdot \left(ACCOA \cdot OAA - \frac{CIT \cdot COA}{Keq} \right)}{KdACCOA0 \cdot KmOAA0}}{\left(\frac{ACCOA}{KdACCOA0} \cdot \left(1 + \frac{AKG}{Ki1AKG} + \frac{NADH}{Ki1NADH} \right) + \frac{ACCOA}{KdACCOA0} \cdot \frac{OAA}{KmOAA0} \cdot \left(1 + \frac{AKG}{Ki2AKG} + \frac{NADH}{Ki2NADH} \right) + \left(1 + \frac{ATP}{KiATP} \right) \cdot \left(1 + \frac{KmACCOA0}{KdACCOA0} \cdot \frac{OAA}{KmOAA0} \right) + \frac{KmcsCOA}{KdcsCOA} \cdot \frac{CIT}{KmcsCIT} + \frac{KmcsCOA}{KdcsCOA} \cdot \frac{ACCOA}{KdACCOA0} \cdot \frac{CIT}{KmcsCIT} + \frac{COA}{KdcsCOA} + \frac{CIT}{KmcsCIT} \cdot \frac{OAA}{KmOAA0} + \frac{ACCOA}{KdACCOA0} \cdot \frac{CIT}{KdcsCIT} \cdot \frac{OAA}{KmOAA0} + \frac{KmACCOA0}{KdACCOA0} \cdot \frac{COA}{KdcsCOA} \cdot \frac{OAA}{KmOAA0} + \frac{CIT}{KmcsCIT} \cdot \frac{COA}{KdcsCOA} \cdot \frac{OAA}{KdcsOAA} \right)}$$

$$vGND = \frac{\frac{Vmax \cdot \left(NADP \cdot PGN - \frac{NADPH \cdot RU5P \cdot HCO3}{Keq} \right)}{KdNADP \cdot KmPGN}}{\left(1 + \frac{NADP}{KdNADP} + \frac{FDP}{KefFbP} + \frac{NADP}{KdNADP} \cdot \frac{FDP}{KefNADPFbP} + \frac{KmNADP}{KdNADP} \cdot \frac{PGN}{KmPGN} + \frac{NADP}{KdNADP} \cdot \frac{PGN}{KmPGN} + \frac{ATP}{KefATP} + \frac{ATP}{KefNADPATP} \cdot \frac{KmNADP}{KdNADP} \cdot \frac{PGN}{KmPGN} + \frac{HCO3}{KdHCO3} + \frac{NADPH}{KdNADPH} + \frac{RU5P}{KdRu5P} + \frac{HCO3}{KdHCO3} \cdot \frac{NADPH}{KdHCO3NADPH} + \frac{HCO3}{KdHCO3} \cdot \frac{KmNADPH}{KdHCO3NADPH} \cdot \frac{RU5P}{KmRU5P} + \frac{HCO3}{KdHCO3} \cdot \frac{NADPH}{KdHCO3NADPH} \cdot \frac{RU5P}{KmRU5P} + \frac{KmHCO3}{KdHCO3} \cdot \frac{NADPH}{KdHCO3NADPH} \cdot \frac{RU5P}{KmRU5P} \right)}$$

$$vGPM=\frac{\frac{Vmax\cdot\left(PGA3-\frac{PGA2}{Keq}\right)}{KmPGA3}}{1+\frac{PGA3}{KmPGA3}+\frac{PGA2}{KmPGA2}}$$

$$vGROWTH=\frac{\frac{Vmax\cdot G6P\cdot E4P\cdot PGA3\cdot OAA\cdot AKG\cdot PYR\cdot R5P\cdot PEP\cdot GAP\cdot F6P\cdot NADPH\cdot ACCOA\cdot NAD\cdot ATP}{KmG6P\cdot KmE4P\cdot KmPGA3\cdot KmOAA\cdot KmAKG\cdot KmPYR\cdot KmR5P\cdot KmPEP\cdot KmGAP\cdot KmF6P\cdot KmNADPH\cdot KmACCOA\cdot KmNAD\cdot KmATP}}{\left(\begin{array}{l}\left(1+\frac{G6P}{KmG6P}\right)\cdot\left(1+\frac{E4P}{KmE4P}\right)\cdot\left(1+\frac{PGA3}{KmPGA3}\right)\cdot\left(1+\frac{OAA}{KmOAA}\right)\cdot\left(1+\frac{AKG}{KmAKG}\right)\cdot\left(1+\frac{PYR}{KmPYR}\right)\cdot\left(1+\frac{R5P}{KmR5P}\right)\\ \cdot\left(1+\frac{PEP}{KmPEP}\right)\cdot\left(1+\frac{GAP}{KmGAP}\right)\cdot\left(1+\frac{F6P}{KmF6P}\right)\cdot\left(1+\frac{NADPH}{KmNADPH}\right)\cdot\left(1+\frac{ACCOA}{KmACCOA}\right)\cdot\left(1+\frac{NAD}{KmNAD}\right)\cdot\left(1+\frac{ATP}{KmATP}\right)\end{array}\right)}$$

$$vICD=\frac{\frac{icd\cdot kcat\cdot\left(ICIT\cdot NADP-\frac{AKG\cdot NADPH}{Keq}\right)}{KmICIT\cdot KmNADP}}{\left(1+\frac{ICIT}{KmICIT}\right)\cdot\left(1+\frac{NADP}{KmNADP}\right)+\left(1+\frac{AKG}{KmAKG}\right)\cdot\left(1+\frac{NADPH}{KmNADPH}\right)-1}$$

$$vLPD=\frac{\frac{Vmax\cdot COA\cdot AKG\cdot NAD\cdot\left(1-\frac{AKG}{KdAKG}\right)}{KmCOA\cdot KmAKG\cdot KmNAD}}{\left(\begin{array}{l}\frac{COA}{KmCOA}\cdot\frac{AKG}{KmAKG}+\frac{COA}{KmCOA}\cdot\frac{NAD}{KmNAD}+\frac{AKG}{KmAKG}\cdot\frac{NAD}{KmNAD}+\frac{COA}{KmCOA}\cdot\frac{AKG}{KmAKG}\cdot\frac{NAD}{KmNAD}\\ -\frac{AKG}{KdAKG}\cdot\left(\frac{COA}{KmCOA}\cdot\frac{AKG}{KmAKG}+\frac{AKG}{KmAKG}\cdot\frac{NAD}{KmNAD}+alpha\cdot\frac{COA}{KmCOA}\cdot\frac{AKG}{KmAKG}\cdot\frac{NAD}{KmNAD}\right)\end{array}\right)}$$

$$vMAE=\frac{\frac{\frac{Vmax\cdot n\cdot MAL\cdot NAD}{KmrMAL\cdot KirNAD}}{1+\frac{KmrNAD}{KirNAD}\cdot\frac{MAL}{KmrMAL}+\frac{NAD}{KirNAD}+\frac{MAL}{KmrMAL}\cdot\frac{NAD}{KirNAD}}\cdot\frac{\frac{MG}{KmrMg}+\frac{MN}{KmrMn}}{1+\frac{MG}{KmrMg}+\frac{MN}{KmrMn}}}{1+L0\cdot\left(\frac{\left(1+\frac{ASP}{KeftASP}\right)\cdot\left(1+\frac{MG}{KmtMg}+\frac{MN}{KmtMn}\right)\cdot\left(1+\frac{ATP}{KeftATP}\right)\cdot\left(1+\frac{ACCOA}{KeftACCOA}+\frac{COA}{KeftCOA}\right)\cdot\left(1+\frac{KmtNAD}{KitNAD}\cdot\frac{MAL}{KmtMAL}+\frac{NAD}{KitNAD}+\frac{MAL}{KmtMAL}\cdot\frac{NAD}{KitNAD}\right)}{\left(1+\frac{ASP}{KefrASP}\right)\cdot\left(1+\frac{MG}{KmrMg}+\frac{MN}{KmrMn}\right)\cdot\left(1+\frac{ATP}{KefrATP}\right)\cdot\left(1+\frac{ACCOA}{KefrACCOA}+\frac{COA}{KefrCOA}\right)\cdot\left(1+\frac{KmrNAD}{KirNAD}\cdot\frac{MAL}{KmrMAL}+\frac{NAD}{KirNAD}+\frac{MAL}{KmrMAL}\cdot\frac{NAD}{KirNAD}\right)}\right)^{n}}$$

$$vMDH=\frac{\frac{Vmax\cdot\left(NADH\cdot OAA-\frac{MAL\cdot NAD}{Keq}\right)}{KiNADH\cdot KmOAA}}{\left(\begin{array}{l}1+\frac{KmNAD}{KiNAD}\cdot\frac{MAL}{KmMAL}+\frac{NAD}{KiNAD}+\frac{MAL}{KmMAL}\cdot\frac{NAD}{KiNAD}+\frac{NADH}{KiNADH}+\frac{KmNAD}{KiNAD}\cdot\frac{MAL}{KmMAL}\cdot\frac{NADH}{KiNADH}+\frac{KmNADH}{KiNADH}\cdot\frac{OAA}{KmOAA}+\frac{KmNADH}{KiNADH}\cdot\frac{NAD}{KiNAD}\cdot\frac{OAA}{KmOAA}\\ +\frac{MAL\cdot NAD\cdot OAA}{KiNAD\cdot KiOAA\cdot KmMAL}+\frac{NADH}{KiNADH}\cdot\frac{OAA}{KmOAA}+Keq\cdot\frac{KiNADH\cdot KmOAA}{KiNAD\cdot KmMAL}\cdot\frac{KmNAD}{KiNAD}\cdot\frac{MAL}{KmMAL}\cdot\frac{NADH}{KmNADH}\cdot\frac{OAA}{KiOAA}\end{array}\right)}$$

$$vMQO=\frac{\frac{Vmax\cdot\left(MAL\cdot Q-\frac{OAA\cdot QH2}{Keq}\right)}{KmMAL\cdot KmQ}}{\left(1+\frac{MAL}{KmMAL}\right)\cdot\left(1+\frac{Q}{KmQ}\right)+\left(1+\frac{OAA}{KmOAA}\right)\cdot\left(1+\frac{QH2}{KmQH2}\right)-1}$$

$$vNDH1=\frac{Vmax}{1+\left(\frac{ln\left(\frac{Hout}{Hin}\right)}{ln10}\right)^{2}}\cdot\left(NADH\cdot Q-\frac{NAD\cdot QH2}{Keq}\right)$$

$$vNDH2=Vmax\cdot\left(NADH\cdot Q-\frac{NAD\cdot QH2}{Keq}\right)$$

$$vPCK=\frac{\frac{Vmax\cdot\left(MgATP\cdot OAA-\frac{HCO3\cdot MgADP\cdot PEP}{Keq}\right)}{KmATP\cdot KmOAA}}{1+\frac{HCO3}{KmHCO3}+\frac{HCO3}{KmHCO3}\cdot\frac{ADP}{KmADP}+\frac{MgADP}{KmADP}+\frac{MgATP}{KmATP}+\frac{OAA}{KmOAA}+\frac{MgATP}{KmATP}\cdot\frac{OAA}{KmOAA}+\frac{HCO3}{KmHCO3}\cdot\frac{PEP}{KmPEP}+\frac{PEP}{KmPEP}+\frac{HCO3}{KmHCO3}\cdot\frac{MgADP}{KmADP}\cdot\frac{PEP}{KmPEP}+\frac{MgADP}{KmADP}\cdot\frac{PEP}{KmPEP}}$$

$$vPDH=\frac{\frac{\frac{Vmax\cdot\left(COA\cdot NAD\cdot PYR-\frac{ACCOA\cdot NADH\cdot HCO3}{Keq}\right)}{KmCOA\cdot KmNAD\cdot KmPYR}}{\left(\begin{array}{l}\frac{ACCOA}{KmACCOA}+\frac{NADH}{KmNADH}+\frac{ACCOA}{KmACCOA}\cdot\frac{NADH}{KmNADH}+\frac{COA}{KmCOA}\cdot\frac{NADH}{KmNADH}+\frac{ACCOA}{KmACCOA}\cdot\frac{COA}{KmCOA}\cdot\frac{NADH}{KmNADH}+\frac{NAD}{KmNAD}\cdot\frac{NADH}{KmNADH}+\frac{COA}{KmCOA}\cdot\frac{NAD}{KmNAD}\cdot\frac{NADH}{KmNADH}\\ +\frac{ACCOA}{KmACCOA}\cdot\frac{PYR}{KmPYR}+\frac{ACCOA}{KmACCOA}\cdot\frac{COA}{KmCOA}\cdot\frac{PYR}{KmPYR}+\frac{COA}{KmCOA}\cdot\left(1+\frac{NAD}{KmNAD}\right)\cdot\frac{PYR}{KmPYR}+\frac{NAD}{KmNAD}\cdot\left(1+\frac{COA}{KmCOA}+\frac{PYR}{KmPYR}\right)\end{array}\right)}}{1+\frac{HCO3}{KmHCO3}}$$

$$vPFK=\frac{\dfrac{\dfrac{Vmax\cdot n\cdot\left(MgATP\cdot F6P-\dfrac{MgADP\cdot FDP}{Keq}\right)}{KirF6P\cdot KmrATPMg}}{\left(\begin{array}{l}1+\dfrac{KmrFDP}{KirFDP}\cdot\dfrac{MgADP}{KmrADP}+\dfrac{KmrF6P}{KirF6P}\cdot\dfrac{MgATP}{KmrATPMg}+\dfrac{KmrFDP}{KirFDP}\cdot\dfrac{MgADP}{KmrADP}\cdot\dfrac{F6P}{KirF6P}+\dfrac{MgATP}{KmrATPMg}\cdot\dfrac{F6P}{KirF6P}+\dfrac{MgADP}{KirADP}\cdot\dfrac{MgATP}{KmrATPMg}\cdot\dfrac{F6P}{KirF6P}\\+\left(1+\dfrac{ATP-MgATP}{KirATP}\right)\cdot\dfrac{F6P}{KirF6P}+\dfrac{FDP}{KirFDP}+\dfrac{MgADP}{KmrADP}\cdot\dfrac{FDP}{KirFDP}+\dfrac{KmrF6P}{KirF6P}\cdot\dfrac{MgATP}{KmrATPMg}\cdot\dfrac{FDP}{KirFDP}+Wr\cdot\dfrac{KmrF6P}{KirF6P}\cdot\dfrac{MgADP}{KirADP}\cdot\dfrac{MgATP}{KmrATPMg}\cdot\dfrac{FDP}{KmrFDP}\end{array}\right)}}{1+L0\cdot\left(\dfrac{\left(\begin{array}{l}1+\dfrac{MgADP}{KeftADP}+\dfrac{PEP}{KeftPEP}\\+\dfrac{MgADP}{KeftADP}\cdot\dfrac{PEP}{KeftPEP}\end{array}\right)\cdot\left(\begin{array}{l}1+\dfrac{KmtFDP}{KitFDP}\cdot\dfrac{MgADP}{KmtADP}+\dfrac{KmtF6P}{KitF6P}\cdot\dfrac{MgATP}{KmtATPMg}+\dfrac{KmtFDP}{KitFDP}\cdot\dfrac{MgADP}{KmtADP}\cdot\dfrac{F6P}{KitF6P}+\dfrac{MgATP}{KmtATPMg}\cdot\dfrac{F6P}{KitF6P}+\dfrac{MgADP}{KitADP}\cdot\dfrac{MgATP}{KmtATPMg}\cdot\dfrac{F6P}{KitF6P}\\+\left(1+\dfrac{ATP-MgATP}{KitATP}\right)\cdot\dfrac{F6P}{KitF6P}+\dfrac{FDP}{KitFDP}+\dfrac{MgADP}{KmtADP}\cdot\dfrac{FDP}{KitFDP}+\dfrac{KmtF6P}{KitF6P}\cdot\dfrac{MgATP}{KmtATPMg}\cdot\dfrac{FDP}{KitFDP}+Wt\cdot\dfrac{KmtF6P}{KitF6P}\cdot\dfrac{MgADP}{KitADP}\cdot\dfrac{MgATP}{KmtATPMg}\cdot\dfrac{FDP}{KmtFDP}\end{array}\right)}{\left(\begin{array}{l}1+\dfrac{MgADP}{KefrADP}+\dfrac{PEP}{KefrPEP}\\+\dfrac{MgADP}{KefrADP}\cdot\dfrac{PEP}{KefrPEP}\end{array}\right)\cdot\left(\begin{array}{l}1+\dfrac{KmrFDP}{KirFDP}\cdot\dfrac{MgADP}{KmrADP}+\dfrac{KmrF6P\cdot MgATP}{KirF6P\cdot KmrATPMg}+\dfrac{KmrFDP}{KirFDP}\cdot\dfrac{MgADP}{KmrADP}\cdot\dfrac{F6P}{KirF6P}+\dfrac{MgATP}{KmrATPMg}\cdot\dfrac{F6P}{KirF6P}+\dfrac{MgADP}{KirADP}\cdot\dfrac{MgATP}{KmrATPMg}\cdot\dfrac{F6P}{KirF6P}\\+\left(1+\dfrac{ATP-MgATP}{KirATP}\right)\cdot\dfrac{F6P}{KirF6P}+\dfrac{FDP}{KirFDP}+\dfrac{MgADP}{KmrADP}\cdot\dfrac{FDP}{KirFDP}+\dfrac{KmrF6P}{KirF6P}\cdot\dfrac{MgATP}{KmrATPMg}\cdot\dfrac{FDP}{KirFDP}+Wr\cdot\dfrac{KmrF6P}{KirF6P}\cdot\dfrac{MgADP}{KirADP}\cdot\dfrac{MgATP}{KmrATPMg}\cdot\dfrac{FDP}{KmrFDP}\end{array}\right)}\right)^{n}}$$

$$vPGI=\frac{\dfrac{Vmax\cdot\left(G6P-\dfrac{F6P}{Keq}\right)}{KmG6P}}{1+\dfrac{F6P}{KmF6P}+\dfrac{G6P}{KmG6P}+\dfrac{PEP}{KmPEP}+\dfrac{PGN}{KmPGN}}$$

$$vPGK=\frac{\dfrac{Vmax\cdot\left(MgADP\cdot BPG-\dfrac{MgATP\cdot PGA3}{Keq}\right)}{KmADPMg\cdot KmBPG}}{1+\dfrac{MgADP}{KmADPMg}+\dfrac{BPG}{KmBPG}+\dfrac{\dfrac{MgADP}{KmADPMg}\cdot BPG}{KmBPG}+\dfrac{MgATP}{KmATPMg}+\dfrac{PGA3}{KmPGA3}+\dfrac{\dfrac{MgATP}{KmATPMg}\cdot PGA3}{KmPGA3}}$$

$$vPGL=\frac{\dfrac{Vmax\cdot\left(GL6P-\dfrac{PGN}{Keq}\right)}{KmGL6P}}{1+\dfrac{GL6P}{KmGL6P}+\dfrac{PGN}{KmPGN}+\dfrac{G6P}{KiG6P}}$$

$$vPIT=Vmax\cdot\left(\frac{\left(\dfrac{ln\left(\dfrac{Hout}{Hin}\right)}{ln10}\right)^{2}}{1+\left(\dfrac{ln\left(\dfrac{Hout}{Hin}\right)}{ln10}\right)^{2}}\cdot\frac{Pp}{KmPp+Pp}-\frac{Kr}{1+\left(\dfrac{ln\left(\dfrac{Hout}{Hin}\right)}{ln10}\right)^{2}}\cdot\frac{P}{KmP+P}\right)$$

$$vPNT=k\cdot\left(NAD\cdot NADPH-\frac{NADH\cdot NADP}{Keq}\right)$$

$$vPPC=\frac{\dfrac{\dfrac{Vmax\cdot n\cdot\left(PEP\cdot HCO3-\dfrac{OAA\cdot P}{Keq}\right)}{KdrPEP\cdot KmrHCO3}}{1+\dfrac{KmrPEP}{KdrPEP}\cdot\dfrac{HCO3}{KmrHCO3}+\dfrac{KmrOAA}{KdrOAA}\cdot\dfrac{P}{KmrP}+\dfrac{OAA}{KdrOAA}+\dfrac{P}{KmrP}\cdot\dfrac{OAA}{KdrOAA}+\dfrac{HCO3}{KmrHCO3}\cdot\dfrac{PEP}{KdrPEP}+\dfrac{PEP}{KdrPEP}}}{1+L0\cdot\left(\dfrac{\begin{array}{l}\left(1+\dfrac{KmtPEP}{KdtPEP}\cdot\dfrac{HCO3}{KmtHCO3}+\dfrac{KmtOAA}{KdtOAA}\cdot\dfrac{P}{KmtP}+\dfrac{OAA}{KdtOAA}+\dfrac{P}{KmtP}\cdot\dfrac{OAA}{KdtOAA}+\dfrac{HCO3}{KmtHCO3}\cdot\dfrac{PEP}{KdtPEP}+\dfrac{PEP}{KdtPEP}\right)\\\cdot\left(1+\dfrac{ACCOA}{KeftACCOA}+\dfrac{FDP}{KeftFDP}+\dfrac{FDP}{KeftFDP}\cdot\dfrac{ACCOA}{KeftFDPACCOA}\right)\cdot\left(1+\dfrac{ASP}{KeftASP}+\dfrac{CYS}{KeftCYS}+\dfrac{CIT}{KeftCIT}+\dfrac{FUM}{KeftFUM}+\dfrac{MAL}{KeftMAL}+\dfrac{SUC}{KeftSUC}\right)\end{array}}{\begin{array}{l}\left(1+\dfrac{KmrPEP}{KdrPEP}\cdot\dfrac{HCO3}{KmrHCO3}+\dfrac{KmrOAA}{KdrOAA}\cdot\dfrac{P}{KmrP}+\dfrac{OAA}{KdrOAA}+\dfrac{P}{KmrP}\cdot\dfrac{OAA}{KdrOAA}+\dfrac{HCO3}{KmrHCO3}\cdot\dfrac{PEP}{KdrPEP}+\dfrac{PEP}{KdrPEP}\right)\\\cdot\left(1+\dfrac{ACCOA}{KefrACCOA}+\dfrac{FDP}{KefrFDP}+\dfrac{FDP}{KefrFDP}\cdot\dfrac{ACCOA}{KefrFDPACCOA}\right)\cdot\left(1+\dfrac{ASP}{KefrASP}+\dfrac{CYS}{KefrCYS}+\dfrac{CIT}{KefrCIT}+\dfrac{FUM}{KefrFUM}+\dfrac{MAL}{KefrMAL}+\dfrac{SUC}{KefrSUC}\right)\end{array}}\right)^{n}}$$

$$vPPS=\frac{\dfrac{Vmax\cdot\left(MgATP\cdot PYR-\dfrac{AMP\cdot PEP\cdot P\cdot MG}{Keq}\right)}{KmATPMg\cdot KmPYR}}{\left(\begin{array}{l}\dfrac{MgATP}{KmATPMg}+alpha\cdot\dfrac{P}{KdP}\cdot\dfrac{MgATP}{KmATPMg}+alpha\cdot\dfrac{AMP}{KdAMP}\cdot\dfrac{MgATP}{KmATPMg}+alpha\cdot\dfrac{P}{KdP}\cdot\dfrac{AMP}{KdAMP}\cdot\dfrac{MgATP}{KmATPMg}+\dfrac{alpha\cdot\dfrac{MG}{KdMg}\cdot\dfrac{P}{KmP}\cdot\dfrac{AMP}{KdAMP}\cdot\dfrac{MgATP}{KdATPMgPPS}}{W\cdot\left(1+\dfrac{MG}{KdMg}\right)}\\+\dfrac{MgATP}{KmATPMg}\cdot\dfrac{AKG}{KefAKG}+\dfrac{\left(1+\dfrac{MG}{KdMg}\right)\cdot\dfrac{AKG}{KefAKG}\cdot\dfrac{PEP}{KmPEP}}{W}+\dfrac{MgATP}{KmATPMg}\cdot\dfrac{OAA}{KefOAA}+\dfrac{\left(1+\dfrac{MG}{KdMg}\right)\cdot\dfrac{OAA}{KefOAA}\cdot\dfrac{PEP}{KmPEP}}{W}+\dfrac{\dfrac{MG}{KdMg}\cdot\dfrac{P}{KmP}\cdot\dfrac{AMP}{KdAMP}}{W}\\+\dfrac{alpha\cdot\dfrac{P}{KdP}\cdot\dfrac{AMP}{KdAMP}\cdot\dfrac{PEP}{KmPEP}}{W}+\dfrac{alpha\cdot\dfrac{MG}{KdMg}\cdot\dfrac{P}{KmP}\cdot\dfrac{AMP}{KdAMP}\cdot\dfrac{PEP}{KmPEP}}{W}+\dfrac{alpha\cdot\left(1+\dfrac{MG}{KdMg}\right)\cdot\left(\dfrac{KmAMP}{KdAMP}\cdot\dfrac{P}{KmP}\cdot\dfrac{PEP}{KmPEP}+\dfrac{AMP}{KdAMP}\cdot\dfrac{PEP}{KmPEP}\right)}{W}\\+\left(1+\dfrac{MG}{KdMg}\right)\cdot\dfrac{PYR}{KmPYR}+\dfrac{MgATP}{KmATPMg}\cdot\dfrac{PYR}{KmPYR}+\dfrac{\dfrac{KdADPMg}{KdMg}\cdot\dfrac{P}{KmP}\cdot\dfrac{MgADP}{KefADP}\cdot\dfrac{AMP}{KdAMP}}{W\cdot\left(1+\dfrac{MG}{KdMg}\right)}+\dfrac{ADP-MgADP}{KefADP}\cdot\dfrac{PYR}{KmPYR}+\dfrac{\dfrac{KdATPMg}{KdMg}\cdot\dfrac{P}{KmP}\cdot\dfrac{AMP}{KdAMP}\cdot\dfrac{MgATP}{KefATP}}{W\cdot\left(1+\dfrac{MG}{KdMg}\right)}\\+\dfrac{ATP-MgATP}{KefATP}\cdot\dfrac{PYR}{KmPYR}+\dfrac{\left(1+\dfrac{MG}{KdMg}\right)\cdot\dfrac{PEP}{KmPEP}}{W}+alpha\cdot\left(1+\dfrac{MG}{KdMg}\right)\cdot\dfrac{PEP}{KdPEP}\cdot\dfrac{PYR}{KmPYR}+\dfrac{\left(1+\dfrac{MG}{KdMg}\right)\cdot\dfrac{PYR}{KdPYR}\cdot\dfrac{PEP}{KmPEP}}{W}\end{array}\right)}$$

$$vPTA=\frac{\dfrac{Vmax\cdot\left(ACCOA\cdot P-\dfrac{ACP\cdot COA}{Keq}\right)}{KiACCOA\cdot KmP}}{1+\dfrac{ACCOA}{KiACCOA}+\dfrac{P}{KiP}+\dfrac{ACP}{KiACP}+\dfrac{COA}{KiCOA}+\dfrac{ACCOA\cdot P}{KiACCOA\cdot KmP}+\dfrac{ACP\cdot COA}{KmACP\cdot KiCOA}}$$

$$vPTS\_0=\frac{kF\cdot ei\cdot PEP^2}{KmPEP^2+PEP^2}-\frac{kR\cdot eiP\cdot PYR^2}{KmPYR^2+PYR^2}$$

$$vPTS\_1=kF\cdot hpr\cdot eiP-kR\cdot hprP\cdot ei$$

$$vPTS\_2=kF\cdot hprP\cdot eiia-kR\cdot hpr\cdot eiiaP$$

$$vPTS\_3=kF\cdot eiicb\cdot eiiaP-kR\cdot eiia\cdot eiicbP$$

$$vPTS\_4=\frac{kF\cdot eiicbP\cdot GLCx}{KmGLC+GLCx}-\frac{kR\cdot eiicb\cdot G6P}{KmG6P+G6P}$$

$$vPYK=\frac{\dfrac{\dfrac{Vmax\cdot n\cdot PEP\cdot MgADP}{KirPEP\cdot KmrADPMg}}{1+\dfrac{KmrPEP}{KirPEP}\cdot\dfrac{MgADP}{KmrADPMg}+\dfrac{MgATP}{KirATP}+\dfrac{MgADP}{KmrADPMg}\cdot\dfrac{PEP}{KirPEP}+\dfrac{KmrADPMg}{KmrADPMg}\cdot\left(1+\dfrac{ADP-MgADP}{KirADP}\right)\cdot\dfrac{PEP}{KirPEP}+\dfrac{PYR}{KirPYR}+\dfrac{MgATP}{KirPyrATP}\cdot\dfrac{PYR}{KirPYR}}}{1+L0\cdot\left(\dfrac{\left(\left(1+\dfrac{KmtPEP}{KitPEP}\cdot\dfrac{MgADP}{KmtADPMg}+\dfrac{MgATP}{KitATP}+\dfrac{MgADP\cdot PEP}{KitPEP\cdot KmtADPMg}+\left(1+\dfrac{ADP-MgADP}{KitADP}\right)\cdot\dfrac{PEP}{KitPEP}+\dfrac{PYR}{KitPYR}+\dfrac{MgATP}{KitPyrATP}\cdot\dfrac{PYR}{KitPYR}\right)\cdot\left(1+\dfrac{SUCCOA}{KeftSUCCOA}+\dfrac{MgATP\cdot SUCCOA}{KeftATP\cdot KeftSUCCOA}\right)\right)}{\left(\left(1+\dfrac{KmrPEP}{KirPEP}\cdot\dfrac{MgADP}{KmrADPMg}+\dfrac{MgATP}{KirATP}+\dfrac{MgADP}{KmrADPMg}\cdot\dfrac{PEP}{KirPEP}+\left(1+\dfrac{ADP-MgADP}{KirADP}\right)\cdot\dfrac{PEP}{KirPEP}+\dfrac{PYR}{KirPYR}+\dfrac{MgATP}{KirPyrATP}\cdot\dfrac{PYR}{KirPYR}\right)\cdot\left(1+\dfrac{FDP}{KefrFDP}+\dfrac{G6P}{KefrG6P}+\dfrac{GL6P}{KefrGL6P}+\dfrac{R5P}{KefrR5P}+\dfrac{RU5P}{KefrRU5P}+\dfrac{S7P}{KefrS7P}+\dfrac{X5P}{KefrX5P}\right)\right)}\right)^n}$$

$$vRPE=\frac{\dfrac{Vmax\cdot\left(RU5P-\dfrac{X5P}{Keq}\right)}{KmRU5P}}{1+\dfrac{RU5P}{KmRU5P}+\dfrac{X5P}{KmX5P}}$$

$$vRPI=\frac{\dfrac{Vmax\cdot\left(RU5P-\dfrac{R5P}{Keq}\right)}{KmRU5P}}{1+\dfrac{RU5P}{KmRU5P}+\dfrac{R5P}{KmR5P}+\dfrac{E4P}{KmE4P}}$$

$$vS7P\_E4P\_TAL=kcat\cdot\left(S7P\cdot tal-\frac{E4P\cdot talC3}{Keq}\right)$$

$$vS7P\_R5P\_TKT=kcat\cdot\left(R5P\cdot tktC2-\frac{S7P\cdot tkt}{Keq}\right)$$

$$vSDH=\frac{\dfrac{Vmax\cdot\left(SUC\cdot Q-\dfrac{FUM\cdot QH2}{Keq}\right)}{KefSUC\cdot KmQ}}{1+\dfrac{FUM}{KefFUM}+\dfrac{KmSUC}{KefSUC}\cdot\dfrac{Q}{KmQ}+\dfrac{KmFUM}{KefFUM}\cdot\dfrac{QH2}{KmQH2}+\dfrac{FUM}{KefFUM}\cdot\dfrac{QH2}{KmQH2}+\dfrac{SUC}{KefSUC}+\dfrac{SUC}{KefSUC}\cdot\dfrac{Q}{KmQ}}$$

$$vSK=\frac{\dfrac{Vmax\cdot\left(ADP\cdot SUCCOA\cdot P-\dfrac{ATP\cdot COA\cdot SUC}{Keq}\right)}{KmADP\cdot KmSUCCOA\cdot KmP}}{\left(1+\dfrac{ADP}{KmADP}\right)\cdot\left(1+\dfrac{SUCCOA}{KmSUCCOA}\right)\cdot\left(1+\dfrac{P}{KmP}\right)+\left(1+\dfrac{ATP}{KmATP}\right)\cdot\left(1+\dfrac{COA}{KmCOA}\right)\cdot\left(1+\dfrac{SUC}{KmSUC}\right)-1}$$

$$vSQR=Vmax\cdot\left(FADH2\cdot Q-\frac{FAD\cdot QH2}{Keq}\right)$$

$$vTPI=\frac{\dfrac{Vmax\cdot\left(DAP-\dfrac{GAP}{Keq}\right)}{KmDAP}}{1+\dfrac{DAP}{KmDAP}+\dfrac{GAP}{KmGAP}}$$

$$vX5P\_GAP\_TKT=kcat\cdot\left(tkt\cdot X5P-\frac{GAP\cdot tktC2}{Keq}\right)$$

$$vXCH\_GLC=\frac{Vmax\cdot\left(\dfrac{GLCx}{KmGLC}-\dfrac{GLCp}{KmGLC}\right)}{1+\dfrac{GLCx}{KmGLC}+\dfrac{GLCp}{KmGLC}}$$

$$vXCH\_P=\frac{Vmax\cdot\left(\dfrac{Px}{KmP}-\dfrac{Pp}{KmP}\right)}{1+\dfrac{Px}{KmP}+\dfrac{Pp}{KmP}}$$

$$vXCH\_ACE1=\frac{Vmax\cdot\left(\dfrac{ACE}{KmACE}-\dfrac{ACEp}{KmACE}\right)}{1+\dfrac{ACE}{KmACE}+\dfrac{ACEp}{KmACE}}$$

$$vXCH\_ACE2=\frac{Vmax\cdot\left(\dfrac{ACEp}{KmACE}-\dfrac{ACEx}{KmACE}\right)}{1+\dfrac{ACEp}{KmACE}+\dfrac{ACEx}{KmACE}}$$

$$vZWF=\frac{\dfrac{Vmax\cdot\left(G6P\cdot NADP-\dfrac{GL6P\cdot NADPH}{Keq}\right)}{KdG6P\cdot KmNADP}}{1+\dfrac{G6P}{KdG6P}+\dfrac{KmG6P}{KdG6P}\cdot\dfrac{NADP}{KmNADP}+\dfrac{G6P}{KdG6P}\cdot\dfrac{NADP}{KmNADP}+\dfrac{KmGL6P}{KdGL6P}\cdot\dfrac{NADPH}{KmNADPH}+\dfrac{GL6P}{KdGL6P}\cdot\dfrac{NADPH}{KmNADPH}}$$

6. Conservation laws

The following equations describe the conservation laws of conserved moieties:

tal_{total} = tal + talC3

tkt_{total} = tkt + tktC2

icd_{total} = icd + icdP

ei_{total} = ei + eiP

$eiia_{total}$ = eiia + eiiaP

$eiicb_{total}$ = eiicb + eiicbP

hpr_{total} = hpr + hprP

Q_{total} = Q + QH_2

NAD_{total} = NAD + NADH

$NADP_{total}$ = NADP + NADPH

FAD_{total} = FAD + $FADH_2$

AxP_{total} = AMP + ADP + ATP + cAMP

7. Concentrations of cofactors and conserved moieties

Concentrations of cofactors, metal ions and conserved moieties were taken from the literature and are given in table S2.

Table S2. Initial intracellular concentrations of cofactors, metal ions and conserved moieties.

Specie	Concentration (mM)	Comment
HCO_3^-	1.4	saturation concentration in water at 298 K, 1 atm, pH=7.5
O_2	0.21	saturation concentration in water at 298 K, 1 atm
$H^+_{cytoplasm}$	3.16×10^{-5}	from [18], $pH_{cytoplasm}$ = 7.5
Mg^{2+}	1	from [21]
Mn^{2+}	0.3	from [22]
Asp	1.17	from [23]
Cys	0.085	from [23]
CoA	0.5	from [24]
AxP_{total}	4.28	from [23]
ICD_{total}	0.043	from [25]
Q_{total}	1	from [24]
TAL_{total}	0.006	from [26]
TKT_{total}	0.007	from [27]
NAD_{total}	1.57	from [3], in agreement with [28]
$NADP_{total}$	0.257	from [3]
FAD_{total}	1	arbitrary

8. Magnesium complexes

The following functions were used to estimate the concentrations of magnesium complexes taking part as substrates in particular enzyme reactions:

$$MgADP = \frac{Mg \cdot ADP}{KdADPMg + Mg}$$

$$MgATP = \frac{Mg \cdot ATP}{KdATPMg + Mg}$$

$$MgFDP = \frac{Mg \cdot FDP}{KdFDPMg + Mg}$$

where MgADP, MgATP and MgFDP are the concentrations of magnesium complexes, ATP, ADP and FDP are the concentrations of free metabolites, Mg is the concentration of free magnesium ions, and KdADPMg, KdATPMg and KdFDPMg are the respective dissociation constants.

9. Model calibration

This section outlines the followed model calibration strategy and lists the values of all the parameters.

To the extent possible, values of the biochemical parameters were taken from the literature. This was the case for 56% of the parameters (253/449, Table S3). Parameters not available in the literature, which do not have a real biochemical meaning (e.g. Michaelis constants of the biomass function), or for which biochemical estimates are generally not indicative of cellular conditions (e.g. Vmax) were estimated to reproduce in the best possible way various experimental data obtained from a unique *E. coli* strain (the model strain K-12 MG1655 wild-type) grown in a unique reference condition (M9 minimal medium, dilution rate = 0.1 h^{-1}, temperature = 37°C, pH = 7.0, pO_2 > 20%). This was critical to prevent biases during parameter estimation since fluxes and metabolite concentrations depends on environmental conditions and strains [29-32]. Experimental data used for parameter estimation were steady state reaction rates and metabolite concentrations [23, 33-35] and time-course concentrations of intracellular metabolites in response to a glucose pulse [23] (S1 Dataset). A total of 276 data points was used to estimate the remaining 196 parameters. Parameter estimation problem was formulated as a constrained optimization problem:

$$\begin{gathered} minimize\ f(p) \\ subject\ to\ g(p) \geq c \end{gathered}$$

where *p* is the parameter vector, *f* is the objective function which evaluates the deviation between the simulated and measured data, *g(p)* is the constraint function vector, and *c* is the constraint vector. The objective function *f* is defined as the sum of squared weighted errors:

$$f(p) = \sum_i \left(\frac{x_i - y_i(p)}{\sigma_i} \right)^2$$

where x_i is the experimental value of the data point *i* with standard deviation σ_i, and $y_i(p)$ is the corresponding simulated value.

Constraints were defined on estimated parameters (10^{-4} mM $\leq K_M \leq 10^3$ mM; 10^{-2} mM/s $\leq Vmax \leq 10^3$ mM/s; $10^{-4} \leq K_{eq} \leq 10^6$) to ensure they are kept within a biologically reasonable range.

The objective function was minimized with the Particle Swarm Optimization algorithm [36] (with a swarm size of 50 and 20,000 iterations), using the Condor-COPASI system [37] on a pool of 2500 CPU cores. Values of all the parameters (and the corresponding reference for values taken from the literature) are listed in Table S3. The experimental and fitted data are provided in S1 Dataset.

Table S3. Parameters of the kinetic model.

Reaction	*Equation*	*Parameter*	*Value*	*Unit*	*Source*
ACEA	[5]	KdICITsuc	0.0049	mM	Estimated
		KdPEP	1.05	mM	[38]
		KdPEPglx	0.0312	mM	Estimated
		KdPEPicit	0.164	mM	Estimated
		KdPGA3	0.8	mM	[38]
		KdSUC	0.53	mM	[38]
		Keq	8.8	1	[38]
		KmGLX	0.13	mM	[38]
		KmICIT	0.063	mM	[38]
		KmSUC	0.59	mM	[38]
		Vmax	1.53	mM/s	Estimated
ACEB	[5]	Keq	230000	1	[39]
		KmACCOA	0.009	mM	[40]
		KmCOA	10	mM	[5]
		KmGLX	0.021	mM	[40]
		KmMAL	15.1	mM	Estimated, in agreement with the value of 10 estimated in [5]
		Vmax	0.353	mM/s	Estimated
ACEK_1	[5]	k	1.25	mM/s	Estimated
		Keq	888	1	Estimated
ACEK_2	[5]	k	0.0332	mM/s	Estimated
		Keq	2000	1	Estimated
ACK	[8]	Keq	174	1	[8]
		KmACE	7	mM	[41]
		KmACP	0.16	mM	[41]
		KmADP	0.5	mM	[41]
		KmATP	0.07	mM	[41]
		Vmax	7.23	mM/s	Estimated
ACN_1	[5]	Keq	0.385	1	Estimated
		KmACO	0.02	mM	[42]
		KmCIT	0.063	mM	Estimated
		KmICIT	9.31	mM	Estimated
		Vmax	9.72	mM/s	Estimated
ACN_2	[5]	Keq	3.5	1	Estimated, in agreement with the value of 2.615 estimated in [5]
		KmACO	0.02	mM	[42]
		KmCIT	0.063	mM	Estimated
		KmICIT	9.31	mM	Estimated
		Vmax	9.87	mM/s	Estimated
ACS	[8]	KmACE	0.07	mM	[43]
		KmATP	0.1	mM	[44]
		KmCOA	0.01	mM	Estimated
		Vmax	7.3	mM/s	Estimated
ADK	see Section 3	k	0.242	mM/s	Estimated
		Keq	0.963	1	Estimated, in agreement with the experimental range of 0.20-1.45 [23]
ATP_NGAM	see Section 3	Keq	3.63	mM	Estimated
		Vmax	1.3	1/s	Estimated
ATP_SYN	see Section 3	Keq	49.8	1	Estimated
		Vmax	109	mM/s	Estimated
CYA	see Section 3	k	0.0041	1/s	Estimated
		KaeiiaP	0.181	mM	Estimated
		Keq	2590	?	Estimated
CYTBO	see Section 3	Keq	12.07	1	Estimated
		Vmax	8.54	mM/s	Estimated
DOS	see Section 3	k	0.0083	1/s	Estimated
		Keq	674	1	Estimated

EDA	[5]	Keq	0.5	mM	[45]
		KmGAP	86.7	mM	Estimated
		KmKDPG	0.06	mM	[46]
		KmPYR	10	mM	[47]
		Vmax	0.0775	mM/s	Estimated
EDD	[5]	Keq	1000	1	[48]
		KmKDPG	0.318	mM	Estimated, in agreement with the value of 1 estimated in [5]
		KmPGN	0.6	mM	[48]
		Vmax	0.111	mM/s	Estimated
ENO	[5]	Keq	3	1	[49]
		KmPEP	0.1	mM	[50]
		KmPGA2	0.1	mM	[50]
		Vmax	11.7	mM/s	Estimated
F6P_E4P_TKT	[5]	kcat	40	mM/s/mM_enz	Estimated, in agreement with the value of 69 estimated in [5]
		Keq	0.5	1	[51]
F6P_GAP_TAL	[5]	kcat	120	mM/s/mM_enz	Estimated, in agreement with the value of 70 estimated in [5]
		Keq	0.11	1	[27]
FBA	[5]	Keq	0.19	mM	[52]
		KmDAP	0.13	mM	[53]
		KmFDP	0.12	mM	[53]
		KmGAP	0.13	mM	[54]
		KiPEP	0.5	mM	[55]
		Vmax	21.7	mM/s	Estimated
FBP	[5]	KdFDPMg	5.81	mM	Estimated, in agreement with the value of 10 estimated in [5]
		KirAMP	0.0012	mM	[56]
		KirAMPFDP	0.256	mM	[56]
		KirF6P	1.12	mM	[56]
		KirF6PMg	0.385	mM	[57]
		KirFDP	1.35	mM	Estimated, in agreement with the value of 1.116 estimated in [5]
		KirFDPMg	0.76	mM	[56-59]
		KirFDPMgMg	0.356	mM	[56-59]
		KirP	3.16	mM	[57]
		KirPF6P	6.6	mM	[57]
		KirPF6PMg	48.4	mM	[57]
		KirPMg	0.856	mM	[57]
		KitAMP	0.000255	mM	[56-59]
		KitAMPFDP	690	mM	[56-59]
		KitF6P	0.304	mM	[57]
		KitF6PMg	315	mM	[57]
		KitFDP	0.043	mM	Estimated
		KitFDPMg	0.00642	mM	[56-59]
		KitFDPMgMg	100	mM	[56-59]
		KitP	0.642	mM	[57]
		KitPF6P	0.00689	mM	[56-59]
		KitPF6PMg	16.5	mM	[57]
		KitPMg	539	mM	[57]
		KmrFDP	0.064	?	Estimated
		KmrMg	0.039	?	[56-59]
		KmtFDP	1.0e-05	?	[56-59]
		KmtMg	55	?	[57]
		L0	0.000815	?	[56-59]
		n	4	?	[60]
		Vmax	0.216	?	Estimated
FUMA	[5]	Keq	5	1	[61]
		KmFUM	0.6	mM	[61]
		KmMAL	0.7	mM	[62]
		Vmax	53.3	mM/s	Estimated

GDH	[5]	Keq	20	l/mmol	[49]
		KmBPG	0.2	mM	[63]
		KmGAP	2.47	mM	Estimated
		KmNAD	0.011	mM	Estimated, in agreement with the value of 0.045 estimated in [5]
		KmNADH	3.7	mM	Estimated
		KmP	0.017	mM	Estimated
		Vmax	8.67	mM/s	Estimated
GLT	[5]	KdACCOA0	0.7	mM	[64]
		KdcsCIT	7.38	mM	Estimated
		KdcsCOA	0.00175	mM	Estimated
		KdcsOAA	0.155	mM	Estimated, in agreement with the value of 0.05 estimated in [5]
		Keq	8300	1	[65]
		Ki1AKG	0.015	mM	[65]
		Ki1NADH	0.00033	mM	[65]
		Ki2AKG	0.256	mM	[65]
		Ki2NADH	0.0504	mM	[65]
		KiATP	0.58	mM	[65]
		KmACCOA0	0.12	mM	[64]
		KmcsCIT	1.16	mM	Estimated
		KmcsCOA	0.0001	mM	Estimated
		KmOAA0	0.00123	mM	Estimated
		Vmax	57	mM/s	Estimated
GND	see Section 3	KdHCO3	59	mM	Estimated, in agreement with the value of 100 estimated in [5]
		KdHCO3NADPH	9.72	mM	Estimated
		KdNADP	0.117	mM	[66]
		KdNADPH	0.0034	mM	[67]
		KdRu5P	0.044	mM	[67]
		KefATP	0.065	mM	[67]
		KefFbP	0.013	mM	[67]
		KefNADPATP	0.14	mM	[67]
		KefNADPFbP	0.0052	mM	[67]
		Keq	50	mM	[67]
		KmHCO3	6.4	mM	Estimated, in agreement with the value of 3 estimated in [5]
		KmNADP	0.049	mM	[68], in agreement with the value of 0.015 estimated in [5]
		KmNADPH	68.4	mM	Estimated, in agreement with the value of 100 estimated in [5]
		KmPGN	0.093	mM	[68]
		KmRU5P	45.2	mM	Estimated, in agreement with the value of 100 estimated in [5]
		Vmax	4.08	mM/s	Estimated
GPM	[5]	Keq	0.565	1	Estimated, in agreement with the value of 0.55 estimated in [5]
		KmPGA2	1.91	mM	Estimated
		KmPGA3	0.115	mM	Estimated, in agreement with the value of 0.19 estimated in [5]
		Vmax	11	mM/s	Estimated
GROWTH	see Section 3	KmG6P	1.21		Estimated
		KmF6P	0.366		Estimated
		KmGAP	0.0249		Estimated
		KmR5P	0.0212		Estimated
		KmE4P	1.63		Estimated
		KmPGA3	0.0765		Estimated
		KmPEP	0.458		Estimated
		KmPYR	0.00464		Estimated
		KmOAA	0.0248		Estimated
		KmAKG	5.12		Estimated
		KmACCOA	0.0494		Estimated
		KmNADPH	3.598		Estimated
		KmNAD	2.822		Estimated
		KmATP	0.0468		Estimated
		Vmax	9.74		Estimated

ICD	[5]	kcat	2460	1/s	Estimated
		Keq	28.2	1	Estimated, in agreement with the value of 25.3 estimated in [5]
		KmAKG	0.038	mM	[69]
		KmICIT	0.011	mM	[69]
		KmNADP	0.006	mM	[69]
		KmNADPH	0.000683	mM	Estimated
LPD	[5]	alpha	16.4	?	Estimated
		KdAKG	14.9	mM	Estimated
		KmAKG	0.02	?	[70]
		KmCOA	0.076	?	[71]
		KmNAD	0.098	?	[70]
		Vmax	0.0684	?	Estimated
MAD	[5]	KefrACCOA	1.83	mM	Estimated, higher than 0.57 [5]
		KefrASP	0.362	mM	[5]
		KefrATP	89	mM	[5]
		KefrCOA	0.268	mM	[5]
		KeftACCOA	0.197	mM	[5]
		KeftASP	0.583	mM	[5]
		KeftATP	0.26	mM	[5]
		KeftCOA	0.268	mM	[5]
		KirNAD	0.636	mM	Estimated
		KitNAD	0.99	mM	Estimated
		KmrMAL	0.213	mM	Estimated
		KmrMg	0.192	mM	Estimated
		KmrMn	0.273	mM	Estimated
		KmrNAD	1.37	mM	Estimated
		KmtMAL	0.093	mM	[5]
		KmtMg	2.38	mM	Estimated
		KmtMn	0.41	mM	Estimated
		KmtNAD	0.108	mM	[5]
		L0	19.9	?	[5]
		n	4	?	[5]
		Vmax	6.64	?	Estimated
MDH	[5]	Keq	100000	1	[72]
		KiNAD	0.0233	mM	Estimated
		KiNADH	0.000197	mM	Estimated
		KiOAA	2.46	mM	[72]
		KmMAL	0.86	mM	[72]
		KmNAD	0.64	mM	[72]
		KmNADH	0.003	mM	[72]
		KmOAA	0.001	mM	[72]
		Vmax	6.11	mM/s	Estimated
MQO	[5]	Keq	9	1	[73]
		KmMAL	0.435	mM	[73]
		KmOAA	75.8	mM	Estimated, in agreement with the value of 50 estimated in [5]
		KmQ	0.0414	mM	[73]
		KmQH2	8.78	mM	Estimated
		Vmax	4.62	mmol/s	Estimated
NDHI	see Section 3	Keq	27.6	1	Estimated
		Vmax	23.1	mM/s	Estimated
NDHII	see Section 3	Keq	27.6	1	Estimated
		Vmax	30.8	mM/s	Estimated
PCK	[5]	Keq	1.88	mM	[49]
		KmADP	0.05	mM	[74]
		KmATP	0.06	mM	[74]
		KmHCO3	2.63	mM	Estimated, in agreement with the value of 3 estimated in [5]
		KmOAA	0.67	mM	[74]
		KmPEP	0.07	mM	[74]
		Vmax	8.09	mM/s	Estimated

PDH	[5]	Keq	3140	1	Estimated
		KmACCOA	10.2	mM	[5]
		KmCOA	0.005	mM	[5]
		KmHCO3	0.00545	mM	Estimated
		KmNAD	0.01	mM	[5]
		KmNADH	6.64	mM	Estimated
		KmPYR	2	mM	[5]
		Vmax	961	mM/s	Estimated
PFK	[5]	KefrADP	0.0735	mM	[5]
		KefrPEP	20	mM	[5]
		KeftADP	9	mM	[5]
		KeftPEP	0.26	mM	[5]
		Keq	2000	1	Estimated
		KirADP	55	mM	[5]
		KirATP	2.5e-05	mM	[5]
		KirF6P	1.846	mM	[5]
		KirFDP	0.046	mM	[5]
		KitADP	80	mM	[5]
		KitATP	0.014	mM	[5]
		KitF6P	0.0086	mM	[5]
		KitFDP	50.5	mM	[5]
		KmrADP	0.69	mM	[5]
		KmrATPMg	8.12e-05	mM	[5]
		KmrF6P	2.05e-05	mM	[5]
		KmrFDP	10	mM	[5]
		KmtADP	2	mM	[5]
		KmtATPMg	3.34	mM	[5]
		KmtF6P	33	mM	[5]
		KmtFDP	10	mM	[5]
		L0	14.09	?	[5]
		n	4	?	[5]
		Vmax	0.185	?	Estimated
		Wr	0.0237	1	Estimated, in agreement with the value of 0.08 estimated in [5]
		Wt	0.147	1	Estimated
PGI	see Section 3	Keq	0.36	1	[23]
		KmF6P	0.147	mM	[75]
		KmG6P	0.28	mM	[75]
		KiPEP	2	mM	[55]
		KiPGN	0.516	mM	Estimated, in agreement with the value of 0.2 estimated in [5]
		Vmax	2.32	mM/s	Estimated
PGK	[5]	Keq	100	1	[49]
		KmADPMg	0.0854	mM	Estimated, in agreement with the value of 0.2 estimated in [5]
		KmATPMg	3.48	mM	Estimated
		KmBPG	0.0113	mM	Estimated, in agreement with the value of 0.018 estimated in [5]
		KmPGA3	2.457	mM	Estimated, in agreement with the value of 1.28 estimated in [5]
		Vmax	16.1	mM/s	Estimated
PGL	[5]	Keq	42.7	1	[5]
		KiG6P	2	mM	Estimated
		KmGL6P	0.023	mM	[5]
		KmPGN	10	mM	[5]
		Vmax	11	mM/s	Estimated
PIT	[5]	Keq	12.2	1	Estimated
		KmP_{per}	0.025	mM	[76]
		KmP_{cyt}	0.1	mM	Estimated
		Vmax	7.15	mM/s	Estimated
PNT	see Section 3	k	2.5	mM/s	Estimated
		Keq	0.182	1	Estimated

PPC	[5]	KdrOAA	4.35	mM	[5]
		KdrPEP	655	mM	[5]
		KdtOAA	17.9	mM	Estimated
		KdtPEP	0.0122	mM	[5]
		KefrACCOA	0.14	mM	[5]
		KefrASP	0.389	mM	Estimated
		KefrCIT	34.4	mM	[5]
		KefrCYS	0.000449	mM	Estimated
		KefrFDP	10	mM	[5]
		KefrFDPACCOA	0.0156	mM	Estimated
		KefrFUM	2.75	mM	[5]
		KefrMAL	0.23	mM	[5]
		KefrSUC	23	mM	[5]
		KeftACCOA	1.28	mM	Estimated
		KeftASP	27.5	mM	Estimated
		KeftCIT	0.522	mM	Estimated
		KeftCYS	0.977	mM	Estimated
		KeftFDP	13.2	mM	Estimated
		KeftFDPACCOA	47.8	mM	Estimated
		KeftFUM	9.76	mM	Estimated
		KeftMAL	0.737	mM	Estimated
		KeftSUC	107	mM	Estimated
		Keq	150	1	Estimated
		KmrHCO3	0.0022	mM	[5]
		KmrOAA	13	mM	Estimated
		KmrP	0.663	mM	Estimated
		KmrPEP	3.2	mM	[5]
		KmtHCO3	0.0022	mM	[5]
		KmtOAA	6.81	mM	Estimated, in agreement with the value of 6.6 estimated in [5]
		KmtP	0.285	mM	Estimated, in agreement with the range of 0.0013-2.1 estimated in [5]
		KmtPEP	5.12	mM	[5]
		L0	6.37E-06	?	[5]
		n	4	?	[5]
		Vmax	21.4	?	Estimated
PPS	[5]	alpha	38900	?	Estimated
		KdADPMg	1.28	?	[5]
		KdAMP	1480	?	[5]
		KdATPMg	0.085	?	[5]
		KdATPMgPPS	0.0549	?	[5]
		KdMg	36.9	mM	[5]
		KdP	346	?	[5]
		KdPEP	95.7	?	[5]
		KdPYR	2740	?	[5]
		KefADP	0.0283	?	[5]
		KefAKG	0.274	?	[5]
		KefATP	0.000628	?	[5]
		KefOAA	0.796	?	[5]
		Keq	2.00E+05	mmol^2/l^2	[5]
		KmAMP	0.000384	?	[5]
		KmATPMg	0.0549	?	[5]
		KmP	85	?	[5]
		KmPEP	20.7	?	[5]
		KmPYR	0.229	?	[5]
		Vmax	0.0164	?	Estimated
		W	10	?	[5]
PTA	[8]	Keq	0.005	1	Estimated
		KiACCOA	0.2	mM	[77]
		KiACP	0.2	mM	[77]
		KiCOA	0.029	mM	[77]
		KiP	13.5	mM	[77]
		KmACP	0.7	mM	[77]
		KmP	6.1	mM	Estimated
		Vmax	2.7	mM/s	Estimated

PTS_0	[4]	kF	12000	?	[4]
		KmPEP	0.6	mM	Estimated, in agreement with the experimental range of 0.2-0.4 [4]
		KmPYR	1	mM	Estimated, in agreement with the experimental range of 1.5-3 [4]
		kR	8000	?	[4]
PTS_1	[4]	k1	200000	l/(mmol*s)	[4]
		k2	8000	l/(mmol*s)	[4]
PTS_2	[4]	k1	61000	l/(mmol*s)	[4]
		k2	47000	l/(mmol*s)	[4]
PTS_3	[4]	k1	11000	l/(mmol*s)	[4]
		k2	4000	l/(mmol*s)	[4]
PTS_4	[4]	kF	4000	?	[4]
		KmG6P	2125	mM	Estimated
		KmGLC	0.02	mM	[4]
		kR	1.0e-05	?	[4]
PYK	[5]	KefrFDP	0.39	mM	[5]
		KeftATP	4.26	mM	[5]
		KeftSUCCOA	9.67	mM	[5]
		KirADP	0.47	mM	[5]
		KirATP	84	mM	[5]
		KirPEP	0.184	mM	[5]
		KirPYR	13.2	mM	[5]
		KirPyrATP	202.6	mM	[5]
		KitADP	0.196	mM	[5]
		KitATP	0.0448	mM	[5]
		KitPEP	0.405	mM	[5]
		KitPYR	0.294	mM	[5]
		KitPyrATP	13.26	mM	[5]
		KmrADPMg	0.358	mM	[5]
		KmrPEP	6.47E-07	mM	[5]
		KmtADPMg	0.0475	mM	[5]
		KmtPEP	0.1	mM	[5]
		L0	50	1	Estimated, in agreement with the value of 25.3 estimated in [5]
		n	4	1	[5]
		Vmax	0.747	mM/s	Estimated
RPE	[5]	Keq	1.5	1	[78]
		KmRU5P	0.872	mM	[78]
		KmX5P	0.893	mM	[78]
		Vmax	6	mM/s	Estimated
RPI	[5]	Keq	0.33	1	[78]
		KmE4P	0.67	mM	[79]
		KmR5P	3.1	mM	[80]
		KmRU5P	4.4	mM	[80]
		Vmax	8	mM/s	Estimated
S7P_E4P_TAL	[5]	kcat	100	l/(mmol*s)	Estimated, in agreement with the value of 35 estimated in [5]
		Keq	26.6	1	[5]
S7P_R5P_TKT	[5]	kcat	200	l/(mmol*s)	Estimated, in agreement with the value of 131 estimated in [5]
		Keq	0.33	1	[5]
SDH	[5]	KefFUM	0.067	mM	[81]
		KefSUC	0.0322	mM	Estimated
		Keq	2250	1	[82]
		KmFUM	1.36	mM	Estimated
		KmQ	0.00161	mM	Estimated, in agreement with the value of 0.002 estimated in [5]
		KmQH2	0.006	mM	Estimated, in agreement with the value of 0.0045 estimated in [5]
		KmSUC	0.806	mM	Estimated
		Vmax	1.56	mM/s	Estimated

SK	[5]	Keq	1.16	1	Estimated
		KmADP	0.00868	mM	Estimated
		KmATP	0.102	mM	Estimated, in agreement with the value of 0.07 estimated in [5]
		KmCOA	0.255	mM	Estimated
		KmP	0.915	mM	Estimated, in agreement with the value of 0.7 estimated in [5]
		KmSUC	0.8	mM	Estimated
		KmSUCCOA	0.0085	mM	Estimated
		Vmax	76.8	mM/s	Estimated
SQR	see Section 3	Keq	0.94	1	Estimated
		Vmax	3.42	mM/s	Estimated
TPI	[5]	Keq	0.27	1	Estimated
		KmDAP	0.01	mM	[5]
		KmGAP	1.89	mM	Estimated
		Vmax	24.2	mM/s	Estimated
X5P_GAP_TKT	[5]	kcat	40	l/(mmol*s)	Estimated
		Keq	1	1	[5]
ZWF	[5]	KdG6P	0.192	mM	[5]
		KdGL6P	0.02	mM	[5]
		Keq	6.00E+10	1	[5]
		KmG6P	0.119	mM	Estimated, in agreement with the value of 0.156 estimated in [5]
		KmGL6P	0.329	mM	Estimated, in agreement with the value of 0.122 estimated in [5]
		KmNADP	0.0274	mM	[5]
		KmNADPH	0.0168	mM	[5]
		Vmax	0.266	mM/s	Estimated

10. Model validation

We first assessed the stability of the model by checking the stability of the Jacobian matrix under two different conditions, namely: the reference state condition (glucose limitation at a growth rate of 0.1 h^{-1}), and glucose excess condition (by fixing extracellular glucose concentration at 10 mM). In both situations the model demonstrates stable steady states with strictly negative Jacobian eigenvalues.

Then, we evaluated the metabolic control analysis results by comparing the predicted flux control to observations. The model predictions were in line with the literature, as detailed in the manuscript.

Finally, we assessed the ability of the model to identify conserved functional couplings that are independent of gene expression. As detailed in the manuscript, we collected 778 flux data from some 266 experiments, where different *E. coli* K-12 wild-type and mutant strains were cultivated under different conditions (in batch, chemostat, or shake flask). It is important to note that these data were not used to calibrate the model, they were used only for validation purpose. This data set is very different from the data set used for parameter estimation, which were from a single *E. coli* strain grown in a unique condition. The 778 data used for validation (growth rates, glucose uptake rates, biomass yields, oxygen uptake rates, and fluxes through the TCA cycle) are provided in Dataset S2. The simulations and measurements are in excellent agreement (Figures 4, 5, and 6 of the manuscript), which indicates the model yielded fairly accurate predictions of the metabolic states that can be expressed by *E. coli* growing on glucose. All the

experimental data support the model-driven hypothesis that metabolic regulation is sufficient to maintain the tight coordination between these key metabolic processes.

11. References

1. Stock, J.B., B. Rauch, and S. Roseman, *Periplasmic space in Salmonella typhimurium and Escherichia coli.* J Biol Chem, 1977. **252**(21): p. 7850-61.
2. Le Novere, N., et al., *The Systems Biology Graphical Notation.* Nat Biotechnol, 2009. **27**(8): p. 735-41.
3. Chassagnole, C., et al., *Dynamic modeling of the central carbon metabolism of Escherichia coli.* Biotechnol Bioeng, 2002. **79**(1): p. 53-73.
4. Rohwer, J.M., et al., *Understanding glucose transport by the bacterial phosphoenolpyruvate:glycose phosphotransferase system on the basis of kinetic measurements in vitro.* J Biol Chem, 2000. **275**(45): p. 34909-21.
5. Peskov, K., E. Mogilevskaya, and O. Demin, *Kinetic modelling of central carbon metabolism in Escherichia coli.* FEBS J, 2012. **279**(18): p. 3374-85.
6. Schreyer, R. and A. Bock, *Phosphoglucose isomerase from Escherischia coli K 10: purification, properties and formation under aerobic and anaerobic condition.* Arch Microbiol, 1980. **127**(3): p. 289-98.
7. Nikolaev, E.V., *The elucidation of metabolic pathways and their improvements using stable optimization of large-scale kinetic models of cellular systems.* Metab Eng, 2010. **12**(1): p. 26-38.
8. Kadir, T.A., et al., *Modeling and simulation of the main metabolism in Escherichia coli and its several single-gene knockout mutants with experimental verification.* Microb Cell Fact, 2010. **9**: p. 88.
9. Mendes, P., S.G. Oliver, and D.B. Kell, *Fitting transporter activities to cellular drug concentrations and fluxes: why the bumblebee can fly.* Trends Pharmacol Sci, 2015. **36**(11): p. 710-23.
10. Borisov, V.B., et al., *Aerobic respiratory chain of Escherichia coli is not allowed to work in fully uncoupled mode.* Proc Natl Acad Sci U S A, 2011. **108**(42): p. 17320-4.
11. Dassa, J., et al., *A new oxygen-regulated operon in Escherichia coli comprises the genes for a putative third cytochrome oxidase and for pH 2.5 acid phosphatase (appA).* Mol Gen Genet, 1991. **229**(3): p. 341-52.
12. Atlung, T. and L. Brondsted, *Role of the transcriptional activator AppY in regulation of the cyx appA operon of Escherichia coli by anaerobiosis, phosphate starvation, and growth phase.* J Bacteriol, 1994. **176**(17): p. 5414-22.
13. Brondsted, L. and T. Atlung, *Effect of growth conditions on expression of the acid phosphatase (cyx-appA) operon and the appY gene, which encodes a transcriptional activator of Escherichia coli.* J Bacteriol, 1996. **178**(6): p. 1556-64.
14. Steigmiller, S., P. Turina, and P. Graber, *The thermodynamic H^+/ATP ratios of the H^+-ATPsynthases from chloroplasts and Escherichia coli.* Proc Natl Acad Sci U S A, 2008. **105**(10): p. 3745-50.
15. Korla, K. and C.K. Mitra, *Modelling the Krebs cycle and oxidative phosphorylation.* J Biomol Struct Dyn, 2014. **32**(2): p. 242-256.
16. Zilberstein, D., et al., *Escherichia coli intracellular pH, membrane potential, and cell growth.* J Bacteriol, 1984. **158**(1): p. 246-52.
17. Slonczewski, J.L., et al., *pH homeostasis in Escherichia coli: measurement by 31P nuclear magnetic resonance of methylphosphonate and phosphate.* Proc Natl Acad Sci U S A, 1981. **78**(10): p. 6271-5.
18. Wilks, J.C. and J.L. Slonczewski, *pH of the cytoplasm and periplasm of Escherichia coli: rapid measurement by green fluorescent protein fluorimetry.* J Bacteriol, 2007. **189**(15): p. 5601-7.
19. Sauer, U., et al., *The soluble and membrane-bound transhydrogenases UdhA and PntAB have divergent functions in NADPH metabolism of Escherichia coli.* J Biol Chem, 2004. **279**(8): p. 6613-9.
20. Feist, A.M., et al., *A genome-scale metabolic reconstruction for Escherichia coli K-12 MG1655 that accounts for 1260 ORFs and thermodynamic information.* Mol Syst Biol, 2007. **3**: p. 121.
21. Froschauer, E.M., et al., *Fluorescence measurements of free [Mg2+] by use of mag-fura 2 in Salmonella enterica.* FEMS Microbiol Lett, 2004. **237**(1): p. 49-55.
22. Hu, Z., et al., *Metal content of metallo-beta-lactamase L1 is determined by the bioavailability of metal ions.* Biochemistry, 2008. **47**(30): p. 7947-53.

23. Taymaz-Nikerel, H., W.M. van Gulik, and J.J. Heijnen, *Escherichia coli responds with a rapid and large change in growth rate upon a shift from glucose-limited to glucose-excess conditions.* Metab Eng, 2011. **13**(3): p. 307-18.
24. Hoque, M.A., et al., *Dynamic responses of the intracellular metabolite concentrations of the wild type and pykA mutant Escherichia coli against pulse addition of glucose or NH3 under those limiting continuous cultures.* Biochemical Engineering Journal, 2005. **26**(1): p. 38-49.
25. Peng, L. and K. Shimizu, *Global metabolic regulation analysis for Escherichia coli K12 based on protein expression by 2-dimensional electrophoresis and enzyme activity measurement.* Appl Microbiol Biotechnol, 2003. **61**(2): p. 163-78.
26. Sprenger, G.A., et al., *Transketolase A of Escherichia coli K12. Purification and properties of the enzyme from recombinant strains.* Eur J Biochem, 1995. **230**(2): p. 525-32.
27. Sprenger, G.A., et al., *Transaldolase B of Escherichia coli K-12: cloning of its gene, talB, and characterization of the enzyme from recombinant strains.* J Bacteriol, 1995. **177**(20): p. 5930-6.
28. Wimpenny, J.W. and A. Firth, *Levels of nicotinamide adenine dinucleotide and reduced nicotinamide adenine dinucleotide in facultative bacteria and the effect of oxygen.* J Bacteriol, 1972. **111**(1): p. 24-32.
29. Phue, J.N., et al., *Glucose metabolism at high density growth of E. coli B and E. coli K: differences in metabolic pathways are responsible for efficient glucose utilization in E. coli B as determined by microarrays and Northern blot analyses.* Biotechnol Bioeng, 2005. **90**(7): p. 805-20.
30. Revelles, O., et al., *The Carbon storage regulator (Csr) system exerts a nutrient-specific control over central metabolism in Escherichia coli strain Nissle 1917.* PLoS One, 2013. **8**(6): p. e66386.
31. Shiloach, J., et al., *Analyzing metabolic variations in different bacterial strains, historical perspectives and current trends--example E. coli.* Curr Opin Biotechnol, 2010. **21**(1): p. 21-6.
32. Waegeman, H., et al., *Effect of iclR and arcA knockouts on biomass formation and metabolic fluxes in Escherichia coli K12 and its implications on understanding the metabolism of Escherichia coli BL21 (DE3).* BMC Microbiol, 2011. **11**: p. 70.
33. Nanchen, A., A. Schicker, and U. Sauer, *Nonlinear dependency of intracellular fluxes on growth rate in miniaturized continuous cultures of Escherichia coli.* Appl Environ Microbiol, 2006. **72**(2): p. 1164-72.
34. Taymaz-Nikerel, H., et al., *Development and application of a differential method for reliable metabolome analysis in Escherichia coli.* Anal Biochem, 2009. **386**(1): p. 9-19.
35. Amin, N. and A. Peterkofsky, *A dual mechanism for regulating cAMP levels in Escherichia coli.* J Biol Chem, 1995. **270**(20): p. 11803-5.
36. Kennedy, J. and R. Eberhart, *Particle Swarm Optimization.* Proceedings of the Fourth IEEE International Conference on Neural Networks, Perth, Australia, 1995: p. 1942-1948.
37. Kent, E., S. Hoops, and P. Mendes, *Condor-COPASI: high-throughput computing for biochemical networks.* BMC Syst Biol, 2012. **6**(1): p. 91.
38. MacKintosh, C. and H.G. Nimmo, *Purification and regulatory properties of isocitrate lyase from Escherichia coli ML308.* Biochem J, 1988. **250**(1): p. 25-31.
39. Alberty, R.A., *Calculating apparent equilibrium constants of enzyme-catalyzed reactions at pH 7.* Biochem Educ, 2000. **28**(1): p. 12-17.
40. Falmagne, P. and J.M. Wiame, *[Purification and partial characterization of two malate synthases of Echerichia coli].* Eur J Biochem, 1973. **37**(3): p. 415-24.
41. Fox, D.K. and S. Roseman, *Isolation and characterization of homogeneous acetate kinase from Salmonella typhimurium and Escherichia coli.* J Biol Chem, 1986. **261**(29): p. 13487-97.
42. Jordan, P.A., et al., *Biochemical and spectroscopic characterization of Escherichia coli aconitases (AcnA and AcnB).* Biochem J, 1999. **344 Pt 3**: p. 739-46.
43. Fung, E., et al., *A synthetic gene-metabolic oscillator.* Nature, 2005. **435**(7038): p. 118-22.
44. Brown, T.D., M.C. Jones-Mortimer, and H.L. Kornberg, *The enzymic interconversion of acetate and acetyl-coenzyme A in Escherichia coli.* J Gen Microbiol, 1977. **102**(2): p. 327-36.
45. Cheriyan, M., E.J. Toone, and C.A. Fierke, *Mutagenesis of the phosphate-binding pocket of KDPG aldolase enhances selectivity for hydrophobic substrates.* Protein Sci, 2007. **16**(11): p. 2368-77.
46. Braga, R., L. Hecquet, and C. Blonski, *Slow-binding inhibition of 2-keto-3-deoxy-6-phosphogluconate (KDPG) aldolase.* Bioorg Med Chem, 2004. **12**(11): p. 2965-72.
47. Wymer, N., et al., *Directed evolution of a new catalytic site in 2-keto-3-deoxy-6-phosphogluconate aldolase from Escherichia coli.* Structure, 2001. **9**(1): p. 1-9.
48. Wood, W.A., *6-Phosphogluconic and related dehydrases.* 3 ed. The Enzymes. 1971.

49. Nelson, D.L. and M.M. Cox, *Lehninger. Principles of Biochemistry. 4th edition*. 2005.
50. Spring, T.G. and F. Wold, *The purification and characterization of Escherichia coli enolase.* J Biol Chem, 1971. **246**(22): p. 6797-802.
51. Datta, A.G. and E. Racker, *Mechanism of action of transketolase. I. Properties of the crystalline yeast enzyme.* J Biol Chem, 1961. **236**: p. 617-23.
52. Babul, J., et al., *Glucose metabolism in Escherichia coli and the effect of increased amount of aldolase.* Biochemistry, 1993. **32**(17): p. 4685-92.
53. Baldwin, S.A., R.N. Perham, and D. Stribling, *Purification and characterization of the class-II D-fructose 1,6-bisphosphate aldolase from Escherichia coli (Crookes' strain).* Biochem J, 1978. **169**(3): p. 633-41.
54. Plater, A.R., et al., *Conserved residues in the mechanism of the E. coli Class II FBP-aldolase.* J Mol Biol, 1999. **285**(2): p. 843-55.
55. Ogawa, T., et al., *Inhibitory effect of phosphoenolpyruvate on glycolytic enzymes in Escherichia coli.* Res Microbiol, 2007. **158**(2): p. 159-63.
56. Kelley-Loughnane, N., et al., *Purification, kinetic studies, and homology model of Escherichia coli fructose-1,6-bisphosphatase.* Biochim Biophys Acta, 2002. **1594**(1): p. 6-16.
57. Fraenkel, D.G., S. Pontremoli, and B.L. Horecker, *The specific fructose diphosphatase of Escherichia coli: properties and partial purification.* Arch Biochem Biophys, 1966. **114**(1): p. 4-12.
58. Babul, J. and V. Guixe, *Fructose bisphosphatase from Escherichia coli. Purification and characterization.* Arch Biochem Biophys, 1983. **225**(2): p. 944-9.
59. Marcus, F., I. Edelstein, and J. Rittenhouse, *Inhibition of Escherichia coli fructose-1,6-bisphosphatase by fructose 2,6-bisphosphate.* Biochem Biophys Res Commun, 1984. **119**(3): p. 1103-8.
60. Parducci, R.E., et al., *Evidence for a catalytic Mg2+ ion and effect of phosphate on the activity of Escherichia coli phosphofructokinase-2: regulatory properties of a ribokinase family member.* Biochemistry, 2006. **45**(30): p. 9291-9.
61. Flint, D.H., *Initial kinetic and mechanistic characterization of Escherichia coli fumarase A.* Arch Biochem Biophys, 1994. **311**(2): p. 509-16.
62. Flint, D.H., M.H. Emptage, and J.R. Guest, *Fumarase a from Escherichia coli: purification and characterization as an iron-sulfur cluster containing enzyme.* Biochemistry, 1992. **31**(42): p. 10331-7.
63. Lambeir, A.M., et al., *The cytosolic and glycosomal glyceraldehyde-3-phosphate dehydrogenase from Trypanosoma brucei. Kinetic properties and comparison with homologous enzymes.* Eur J Biochem, 1991. **198**(2): p. 429-35.
64. Faloona, G.R. and P.A. Srere, *Escherichia coli citrate synthase. Purification and the effect of potassium on some properties.* Biochemistry, 1969. **8**(11): p. 4497-503.
65. Mogilevskaya, E., et al., *Kinetic modeling of E. coli enzymes: Integration of in vitro experimental data*. Systems Biology and Biotechnology of Escherichia coli. 2009.
66. Hino, Y. and S. Minakami, *Hexose-6-phosphate and 6-phosphogluconate dehydrogenases of rat liver microsomes. Involvement in NADPH and carbon dioxide generation in the luminal space of microsomal vesicles.* J Biochem, 1982. **92**(2): p. 547-57.
67. de Silva, A.O. and D.G. Fraenkel, *The 6-phosphogluconate dehydrogenase reaction in Escherichia coli.* J Biol Chem, 1979. **254**(20): p. 10237-42.
68. Chen, Y.Y., et al., *Conformational changes associated with cofactor/substrate binding of 6-phosphogluconate dehydrogenase from Escherichia coli and Klebsiella pneumoniae: Implications for enzyme mechanism.* J Struct Biol, 2010. **169**(1): p. 25-35.
69. Mogilevskaia, E.A., et al., *[A kinetic model of functioning and regulation of Escherichia coli isocitrate dehydrogenase].* Biofizika, 2007. **52**(1): p. 47-56.
70. Waskiewicz, D.E. and G.G. Hammes, *Elementary steps in the reaction mechanism of the alpha-ketoglutarate dehydrogenase multienzyme complex from Escherichia coli: kinetics of succinylation and desuccinylation.* Biochemistry, 1984. **23**(14): p. 3136-43.
71. Amarasingham, C.R. and B.D. Davis, *Regulation of alpha-ketoglutarate dehydrogenase formation in Escherichia coli.* J Biol Chem, 1965. **240**(9): p. 3664-8.
72. Wright, S.K., et al., *Mechanistic studies on malate dehydrogenase from Escherichia coli.* Arch Biochem Biophys, 1995. **321**(2): p. 289-96.
73. Molenaar, D., M.E. van der Rest, and S. Petrovic, *Biochemical and genetic characterization of the membrane-associated malate dehydrogenase (acceptor) from Corynebacterium glutamicum.* Eur J Biochem, 1998. **254**(2): p. 395-403.

74. Wright, J.A. and B.D. Sanwal, *Regulatory mechanisms involving nicotinamide adenine nucleotides as all teric effectors. II. Control of phosphoenolpyruvate carboxykinase.* J Biol Chem, 1969. **244**(7): p. 1838-45.
75. Gao, H. and J.A. Leary, *Kinetic measurements of phosphoglucomutase by direct analysis of glucose-1-phosphate and glucose-6-phosphate using ion/molecule reactions and Fourier transform ion cyclotron resonance mass spectrometry.* Anal Biochem, 2004. **329**(2): p. 269-75.
76. Rosenberg, H., R.G. Gerdes, and K. Chegwidden, *Two systems for the uptake of phosphate in Escherichia coli.* J Bacteriol, 1977. **131**(2): p. 505-11.
77. Campos-Bermudez, V.A., et al., *Functional dissection of Escherichia coli phosphotransacetylase structural domains and analysis of key compounds involved in activity regulation.* FEBS J, 2010. **277**(8): p. 1957-66.
78. Horecker, B.L. and J. Hurwitz, *The purification of phosphoketopentoepimerase from Lactobacillus pentosus and the preparation of xylulose 5-phosphate.* J Biol Chem, 1956. **223**(2): p. 993-1008.
79. Woodruff, W.W., 3rd and R. Wolfenden, *Inhibition of ribose-5-phosphate isomerase by 4-phosphoerythronate.* J Biol Chem, 1979. **254**(13): p. 5866-7.
80. Essenberg, M.K. and R.A. Cooper, *Two ribose-5-phosphate isomerases from Escherichia coli K12: partial characterisation of the enzymes and consideration of their possible physiological roles.* Eur J Biochem, 1975. **55**(2): p. 323-32.
81. Maklashina, E. and G. Cecchini, *Comparison of catalytic activity and inhibitors of quinone reactions of succinate dehydrogenase (Succinate-ubiquinone oxidoreductase) and fumarate reductase (Menaquinol-fumarate oxidoreductase) from Escherichia coli.* Arch Biochem Biophys, 1999. **369**(2): p. 223-32.
82. Hirsch, C.A., et al., *A fumarate reductase in Escherichia coli distinct from succinate dehydrogenase.* J Biol Chem, 1963. **238**: p. 3770-4.
